# Pyrazine derivative synthesis in a continuous-flow system: a green synthesis of pyrazinamide from pyrazine esters and amines catalyzed by Lipozyme® TL IM from *Thermomyces lanuginosus*†

**DOI:** 10.1039/d4ra06761d

**Published:** 2024-12-16

**Authors:** Ao-Ying Zhang, Zong-Hao Huang, Li-Hua Du, Hang Lin, Han-Jia Xie, Bing-Lin Yan, Miao Miao Xue, Lin Wang, Wen-Xuan Shao, Guo-Neng Fu, Xi-Ping Luo

**Affiliations:** a College of Pharmaceutical Science, ZheJiang University of Technology Zhejiang Hangzhou 310014 China orgdlh@zjut.edu.cn +86-571-88320903 +86-189-690-693-99; b Zhejiang Provincial Key Laboratory of Chemical Utilization of Forestry Biomass, Zhejiang A&F University Zhejiang Hangzhou 311300 China

## Abstract

Pyrazinamide derivatives have been extensively studied for their biological activities, such as anti-tuberculosis activity and antiviral activities. In this work, a continuous-flow system was developed for the synthesis of pyrazinamide derivatives from pyrazine esters and amines (aliphatic amine, benzylamines and morpholine) catalyzed by Lipozyme® TL IM from *Thermomyces lanuginosus*, which was used for the first time. The reaction parameters including solvent, substrate ratio, reaction temperature and reaction time/flow rate were also studied in detail. A total of 23 pyrazinamide derivatives can be obtained through this method in parallel. Compared with other works, this method can be conducted at 45 °C for 20 min in a greener *tert*-amyl alcohol solvent and maximum yield (91.6%) was obtained as well. In brief, a more efficient and greener method for the synthesis of pyrazinamide derivatives was developed with good scalability, various substrates including aliphatic amines, benzylamines and morpholines can be applied to this method and achieve a desirable yield. Through the construction and research of amide bonds, this method provides a greener and more efficient biocatalytic continuous technology for the development of pyrazine-derived drugs, and provides a basis for the rapid synthesis of pyrazine-derived drugs in the future.

## Abstract 2

The continuous flow synthesis of a series of pyrazinamide derivatives catalyzed by Lipozyme® TL IM was successfully achieved with good scalability. A continuous production of pyrazinamide derivatives was first performed coupled with enzymes.

## Graphical abstract

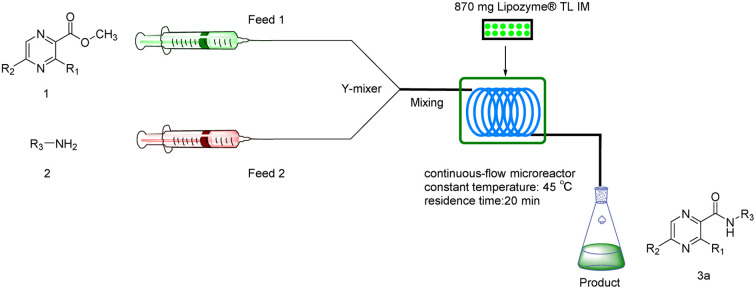

## Introduction

**Fig. 1:**
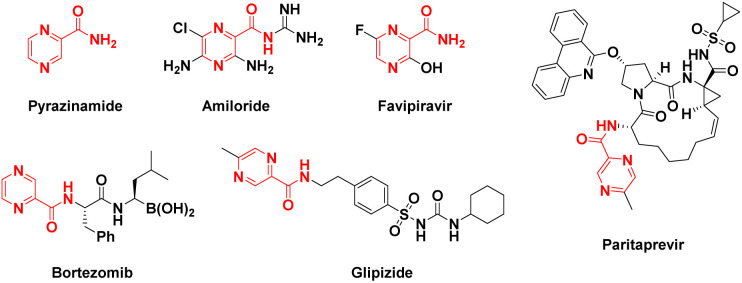
Structures of clinically used pyrazine-derived drugs.

Pyrazines and pyrazine-based compounds are widely spread in nature, and the pyrazine core is a component of many biologically active compounds.^1–4^ Pyrazine derivatives also continue to attract the attention of several investigators due to their diverse biological applications like antifungal,^5^ antitubercular,^6,7^ antiviral,^8^ anti-inflammatory,^9^ anticancer,^10,11^ antiparasitic,^12,13^ anti-malaria^14^ and so forth. Many drugs containing the pyrazine structure have been used in clinical therapy, such as pyrazinamide (antitubercular),^15^ amiloride (diuretics),^16^ favipiravir (antiviral),^17^ bortezomib (proteasome inhibitor),^18^ glipizide (hypoglycemic drug),^19^ and paritaprevir (NS3-4A serine protease inhibitor)^20^ (Fig. 1).

**Fig. 2:**
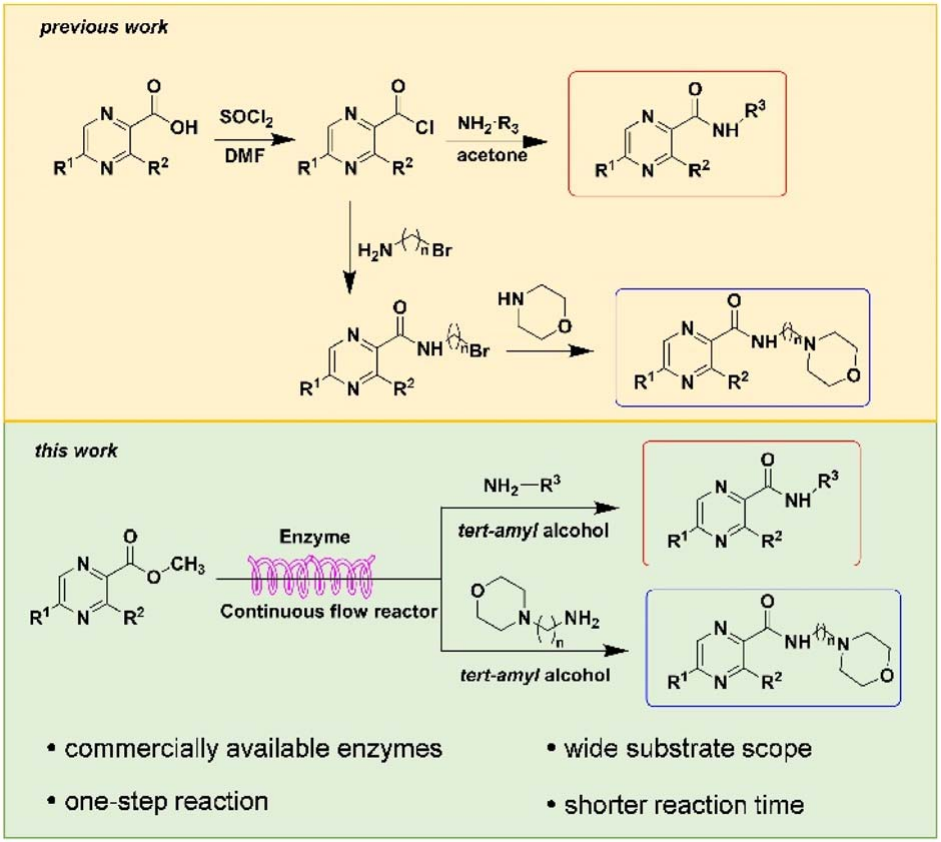
Synthetic routes to pyrazinamide derivatives.

Construction of amide bonds is one of the key steps to obtain pyrazine-derived drugs. The importance of amides has promoted the development of efficient methodologies for amide synthesis.^21,22^ The most widely used methods rely on coupling of amine with carboxylic acids and carboxylic acid derivatives.^23^ Amide synthesis is prevalent in both small and large-scale processes, among the reported methods for the synthesis pyrazinamide derivatives, the most commonly used methods involve generating pyrazinyl chloride from thionyl chloride in DMF (*N*,*N*-dimethylformamide) solvent, followed by a series of reactions to produce pyrazinamide from pyrazine acid.^24–27^ Another viable route is to use pyrazine acid in THF (tetrahydrofuran) solvent, utilizing activating agents like DMAP (4-dimethylaminopyridine) or trifluoroethanol (Fig. 2).^28,29^ The dipolar aprotic solvents used in those methods have reprotoxicity concerns, and the use of those activating reagents always leads to the generation of often toxic or hazardous by-products, alongside increases costs associated with their disposal. It is therefore perhaps unsurprising that more atom economical and preferably catalytic methods for amide bond formation were voted number one on the list of desirable transformations by the American Chemical Society Green Chemistry Institute.^30^

Biocatalytic approaches to amide bond synthesis have proven of intense interest to the chemistry community.^31^ Enzyme is produced from readily available renewable resources and is biodegradable and essentially nonhazardous and nontoxic. As shown by Daniela Manova *et al.*, immobilized *Candida Antarctica* Lipase B (CAL-B) is reported for enzymatic amidation conditions which are compatible with a wide range of amines and acids.^32^ Enzymes that can also catalyse the direct amidation of acids and amines include McbA (ATP-dependent amide bond synthetase from *Marinactinospora thermotolerans*),^33^ SfaB (an acyl adenylate-forming enzyme),^34^ truncated carboxylic acid reductases,^35^ and the combined use of *N*-acyltransferases and CoA ligases.^36^ Intracellular SpL from *Sphingomonas* sp. HXN-200 and Lipozyme® TL IM from *Thermomyces lanuginosus* can be used to the amidation of carboxylic esters and amines.^37,38^ The enzymatic method provides a valuable starting point, however, it presents certain limitations. For instance, the cofactors necessary for the activity of McbA are excessively costly, restricting its applicability in industrial settings, and other enzymes employed in similar contexts also entail substantial expenses. Few studies have been reported on the synthesis of pyrazinamide derivatives through enzyme catalytic reactions, so we envisioned a one-step catalytic synthesis of pyrazinamide using a commercially available and low-cost Lipozyme (Lipozyme® TL IM). This method not only simplifies the steps of a reaction but also minimizes the use of hazardous reagents, in line with the principles of green chemistry. Although, enzymatic reaction conditions are mild, they are associated with long reaction time (20 h or more) and the use of potentially toxic or hazardous reagents.^39^ The application of continuous-flow system can favourably influence the sustainability of the reaction.^40^ The small-sized channel reactors enable dramatically increased mass- and heat-transfer processes, thus assuring high efficiency and controllability in the reaction.^41^ Therefore, we envisage that pyrazinamide can be enzymatically synthesized efficiently and cost-effectively in a continuous flow system.

**Scheme 1:**
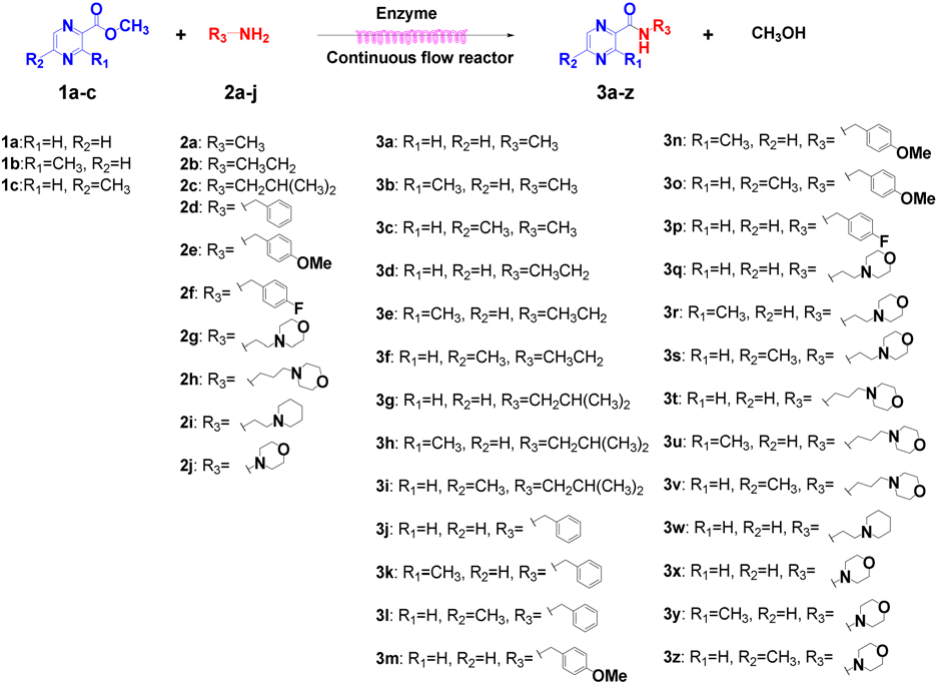
Synthesis of pyrazinamide derivatives in the continuous flow microreactors.

Continuous flow processing is now widely accepted as a disruptive technology in the synthesis of active pharmaceutical ingredients (APIs) as well as other fine and commodity chemicals,^42–44^ continuous flow microreactors have become mainstream heating sources in contemporary pharmaceutical company laboratories.^45^ In early 2019, the IUPAC organization named flow chemistry among the top ten emerging technologies in chemistry and the FDA declared continuous manufacturing (CM) as one of the most important tools in the modernization of the pharmaceutical industry.^46,47^ Compared to batch reactions, continuous flow presents significant advantages, including a high ratio of surface-to-volume, enhanced heat transfer and precise temperature control, higher reaction rates, good control of the residence time, easy automation and scale-up and cost effectiveness.^48,49^ Inspired by the important role of pyrazine drugs in clinical therapy and the urgent need for construction of amide bonds in pyrazine drugs, we have studied the aminolysis reactions from pyrazine esters (pyrazine-2-carboxylate, methyl 3-methylpyrazine-2-carboxylate, methyl 5-mathylpyrazine-2-carboxylate) and several aliphatic amines (methylamine, ethylamine, isobutylamine), benzylamines (benzylamine, 4-methoxybenzylamine) and morpholine (*N*-aminomorpholine, *N*-(2-aminoethyl)morpholine, *N*-(3-aminopropyl)morpholine) in a continuous-flow system catalyzed by Lipozyme® TL IM from *Thermomyces lanuginosus* (Scheme 1), and the batch reactions were also used for comparison. Reaction parameters such as solvent, substrate ratio, reaction temperature and reaction time/flow rate were examined, the influence of reactant structure on pyrazinamide derivatives synthesis reaction had also been studied. A series of different pyrazinamide derivatives were synthesized efficiently with high yields in this continuous-flow enzymatic strategy.

## Results and discussion

### Effect of reaction solvent and enzyme

The use of enzymes in organic solvents for chemical biosynthesis offers a number of potential advantages, as the nature of the solvent can have a profound effect on the substrate specificity, regioselectivity and enantioselectivity of the enzyme. In general, solvents are selected based on their hydrophobicity (log *P*) value, which ranges from 0.6 to 3.5, as the activity and stability of enzyme can be maximum in this range.^50^ The ammonolysis reaction of pyrazine-2-carboxylate and benzylamine was carried out using Lipozyme® TL IM from *Thermomyces lanuginosus* in the continuous flow reactors. We performed a blank control experiment without any enzyme and found that only trace amounts of product were generated. The effect of various organic solvents on the synthetic reaction was studied and the solvents used were methanol, ethanol, isopropanol, isobutanol, *tert*-amyl alcohol, acetonitrile, dichloromethane, DMSO, THF and 2-MeTHF. The log *P* values of the solvents used in the experiment and yields of product are shown in the Table 1. As shown in Table 1, the product yield was higher when *tert*-amyl alcohol was used as the solvent, while by-products such as pyrazine esters were produced in ethanol, isopropanol, and isobutanol. The yield was lower in methanol, acetonitrile, dichloromethane, DMSO, THF and 2-MeTHF. Therefore, *tert*-amyl alcohol was selected as the reaction solvent for further research on the enzymatic synthesis of pyrazinamide derivatives in continuous-flow microreactors. As shown in Table 2, we conducted an experiment on the direct aminolysis of 2-pyrazinecarboxylic acid and benzylamine to synthesize pyrazinamide (3j) catalysed by CAL-B, and the yield of pyrazinamide (3j) was 73.2%. Therefore, we chose Lipozyme® TL IM as the catalyst.

**Table 1:** The effect of reaction media on the enzymatic synthesis of pyrazinamide derivatives in continuous-flow microreactors^a^

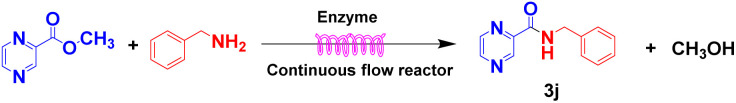				
Entry	Solvent	Catalysts	Log *P*	Yield^b^ (%)
1	*tert*-Amyl alcohol	None	1.04	Trace
2	Methanol	Lipozyme® TL IM	−0.76	54.6 ± 1.3
3	Ethanol	Lipozyme® TL IM	−0.19	35.7 ± 0.9
4	Isopropanol	Lipozyme® TL IM	−0.39	23.6 ± 1.1
5	Isobutanol	Lipozyme® TL IM	0.69	27.8 ± 1.2
6	*tert*-Amyl alcohol	Lipozyme® TL IM	1.04	77.2 ± 0.7
7	Acetonitrile	Lipozyme® TL IM	−0.33	57.9 ± 0.6
8	Dichloromethane	Lipozyme® TL IM	1.19	56.2 ± 1.1
9	DMSO	Lipozyme® TL IM	−1.35	10.3 ± 1.4
10	THF	Lipozyme® TL IM	0.45	63.2 ± 0.7
11	2-MeTHF	Lipozyme® TL IM	1.01	74.1 ± 1.0

a Experimental conditions: in the continuous flow reactors, feed 1, 10 mL solvent contained 5.0 mmol pyrazine-2-carboxylate; feed 2, 10 mL solvent contained 20.0 mmol benzylamine, 45 °C, flow rate 31.2 μL min^−1^ residence time 20 min, enzyme 870 mg.

b Isolated yield. Yield: 100 × (actual received amount/ideal calculated amount). The data are presented as average ± standard deviation (SD) of triplicate experiments.

**Table 2:** The effect of enzymes on the synthesis of pyrazinamide (3j) in continuous-flow microreactors^a^

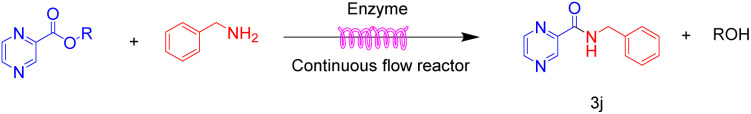				
Entry	Method	R	Enzyme	Yield^b^ (%)
1	A	CH_3_	Lipozyme® TL IM	77.2 ± 0.7
2	B	H	CAL-B	73.2 ± 0.9

a General experimental conditions: Method A: continuous flow reactors, feed 1, dissolve 5 mmol of pyrazine-2-carboxylate in 10 mL *tert*-amyl alcohol; feed 2, dissolve 15 mmol of benzylamine in 10 mL *tert*-amyl alcohol, flow rate 31.2 μL min^−1^, residence time 20 min, enzyme 870 mg, 45 °C. Method B: continuous flow reactors, feed 1, dissolve 5 mmol of 2-pyrazinecarboxylic acid in 10 mL *tert*-amyl alcohol; feed 2, dissolve 15 mmol of benzylamine in 10 mL *tert*-amyl alcohol, flow rate 31.2 μL min^−1^, residence time 20 min, enzyme 870 mg, 45 °C.

b Isolated yield. Yield: 100 × (actual obtained amount/calculated amount). The data are presented as average ± SD of triplicate experiments.

### Effect of substrate ratio

**Fig. 3:**
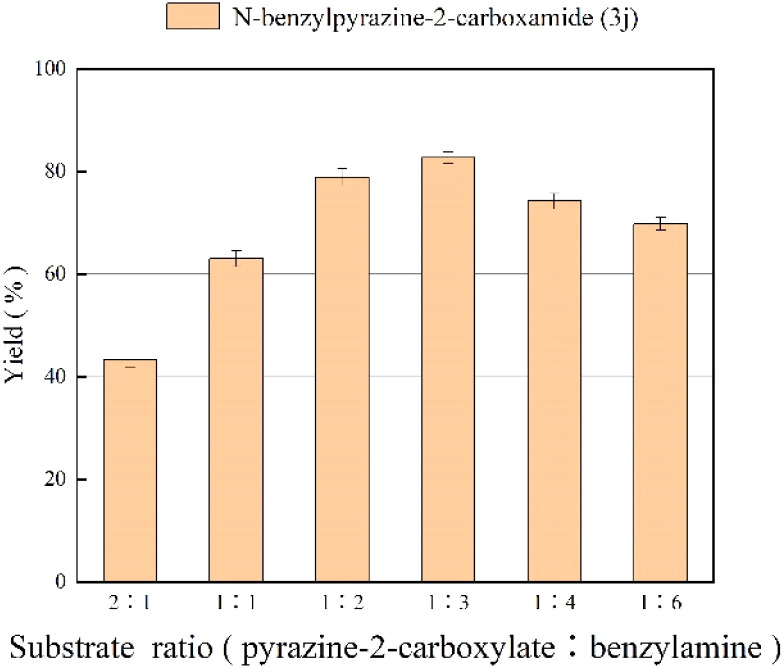
The effect of substrate ratio (pyrazine-2-carboxylate : benzylamine) on the enzymatic synthesis of pyrazinamide derivatives in continuous-flow microreactors.

The effect of mole ratio of substrates on amidation reaction is shown in Fig. 3. From Fig. 3, we can find that the yield of *N*-benzylpyrazine-2-carboxamide increases gradually with the increase of the ratio of benzylamine, which is due to the fact that the amidation reaction is reversible. The highest yield of 81.7% was obtained when the substrate molar ratio (pyrazine-2-carboxylate : benzylamine) was 1 : 3. However, when the molar ratio was increased further, the yield decreases because enzyme activity can be inhibited by excessive amounts of substrate, and substrate concentration that is too high increases viscosity, makes the interaction between reactants become ineffective and inhibits the reaction.^51^ Hence, the substrate ratio of 1 : 3 was chosen as the optimal ratio.

### Effect of reaction temperature

**Fig. 4:**
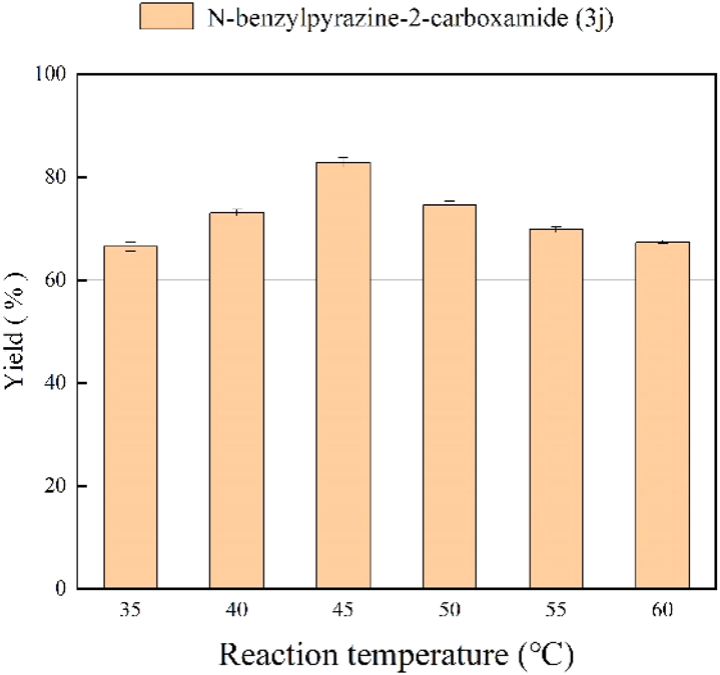
The effect of reaction temperature on the enzymatic synthesis of pyrazinamide derivatives in continuous-flow microreactors.

In the enzymatic reaction, temperature has a large effect on enzyme activity and stability as well as on the rate of reaction. In *tert*-amyl alcohol solvent, we adjusted the reaction temperature from 35 °C to 55 °C, and the results at different temperatures with 35 min residence time are shown in Fig. 4. At higher temperatures the enzymatic reaction will be faster because the solubility of the substrate increases and the collision between particles is rapid.^52^ The best yield of 81.2% is obtained when the reaction temperature is 45 °C, a further increase in temperature might result in irreversible denaturation and inactivation of enzyme, thus resulting in a decrease in yield.^53^ Therefore, we chose 45 °C as the optimal reaction temperature.

### Effect of residence time

**Fig. 5:**
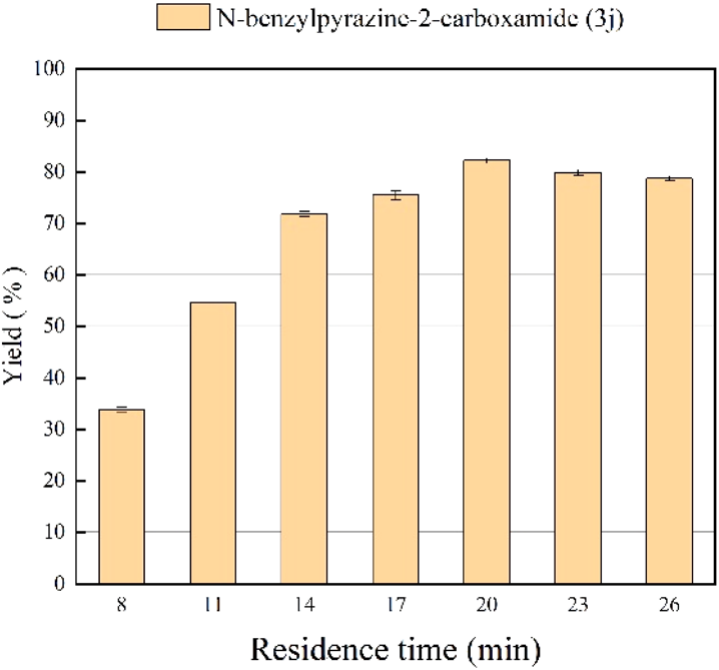
The effect of residence time on the synthesis of pyrazinamide derivatives in continuous-flow microreactors.

The influence of residence time on the yield of pyrazinamide was also studied. In order to obtain a high conversion rate, it is very important that there should be appropriate contact time between enzyme active site and substrate molecule which can be maintained at a specific residence time. In the experiments, the residence time is controlled by adjusting the flow rate while keeping the length of the microbore tube. As can be seen in Fig. 5, the best yield of 82.2% is reached in 20 min (flow rate of 31.2 μL min^−1^), and continuing to prolong the reaction time does not significantly improve the reaction yield. Thus, 20 min was selected as the optimal residence time for the following studies.

### The effect of enzyme reusability

**Fig. 6:**
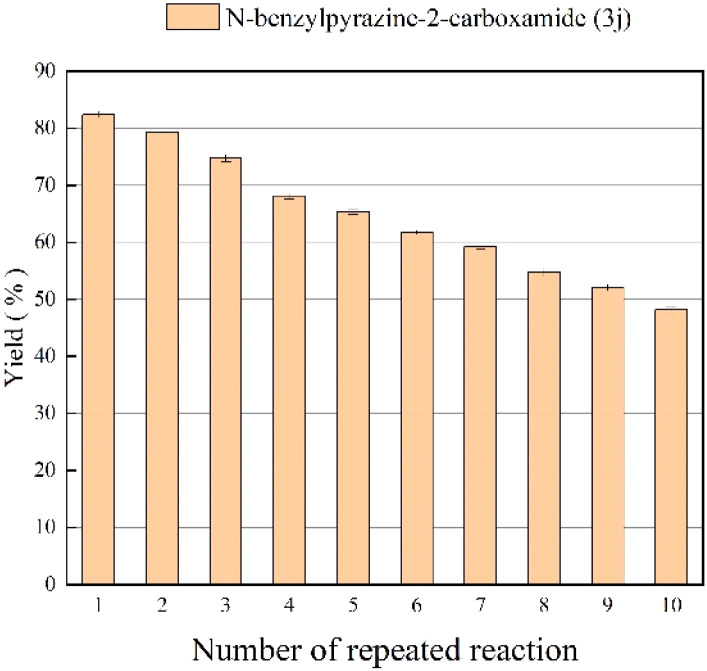
The effect of enzyme reusability on the enzymatic synthesis of nicotinamide derivatives in continuous-flow microreactors.

Since reuse of immobilized lipase reduces production costs, we tested the reusability of Lipozyme® TL IM from *Thermomyces lanuginosus* in the continuous-flow reactor. After 10 catalytic cycles of the same enzyme sample, it was found that the catalytic yield of the last catalysis could still reach 48.7% (Fig. 6). The short reaction time and the excellent reuse potential of the lipozyme may lead to significant productivity gains in the synthesis of pyrazinamide derivatives.

### Comparing the synthesis of pyrazinamide derivatives from pyrazine-2-carboxylate and benzylamine in a continuous-flow microreactor and a shaker reactor

Then, to explore the effect of enzymatic reactions in continuous-flow systems and batch systems, space–time yield (STY) is used as an indicator. As shown in Table 3, the STY in the continuous-flow microreactor was higher and the choice of continuous-flow microreactors is more favorable to improve the efficiency of enzymatic synthesis of pyrazinamide derivatives. In the traditional shaker reactor (Method B), the enzymatic reaction took about 17 hours to reach the expected yield. However, in the continuous-flow microreactor, better yields were obtained in 20 min (Method A).
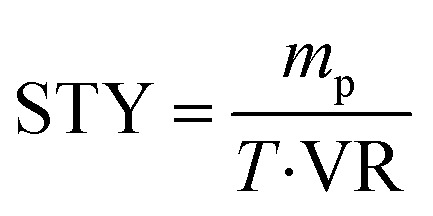
where *m*_p_ is the mass of the generated product (g), *T* is the residence time (h), and VR is the reactor volume (L).

**Table 3:** Enzymatic synthesis of pyrazinamide derivatives in the continuous-flow microreactor or the shaker reactor^a^

Entry	Method	STY (g L^−1^ h^−1^)	Yield^b^ (%)
1	A	263.25	82.3 ± 1.2
2	B	0.92	73.6 ± 0.9

a General experimental conditions: Method A: continuous flow reactors, feed 1, dissolve 5 mmol of pyrazine-2-carboxylate in 10 mL *tert*-amyl alcohol; feed 2, dissolve 15 mmol of benzylamine in 10 mL *tert*-amyl alcohol, flow rate 31.2 μL min^−1^, residence time 20 min, enzyme 870 mg, 45 °C. Method B: shaker reactors, add 5 mmol of pyrazine-2-carboxylate, 15 mmol of benzylamine and 20 mL *tert*-amyl alcohol to a 50 mL erlenmeyer flask, enzyme 870 mg, 160 rpm, 45 °C, 17 h.

b Isolated yield. Yield: 100 × (actual obtained amount/calculated amount). The data are presented as average ± SD of triplicate experiments.

### The scope and limitation of the synthesis of pyrazinamide derivatives catalyzed by lipozyme TL IM in continuous-flow microreactors

Then, the scope and limitations of biocatalytic aminolysis of pyrazinamide derivatives are explored, and the experiments are processed in the continuous flow microreactor and shaker reactors under optimal reaction conditions. As shown in Table 4, the yield of ammonolysis reaction with aliphatic amine (*e.g.*, entry 1, 86.4%) was higher than which with benzylamine (*e.g.*, entry 10, 80.3%). Meanwhile, benzylamine with an electron-donating group (*e.g.*, entry13, 83.5%) were more favourable for the reaction than benzylamine with an electron-withdrawing group (*e.g.*, entry 16, 67.7%).

**Table 4:** The effect of amines structure to the enzymatic synthesis of pyrazinamide derivatives under continuous-flow conditions^a^

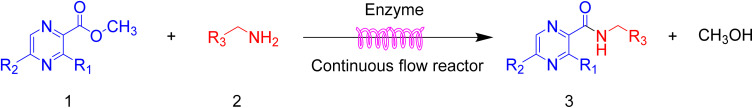							
Entry	R_1_	R_2_	R_3_	Method	Time	Product	Yield^b^ (%)
1	H	H	H	A	20 min	3a	86.4 ± 0.7
				B	17 h		79.7 ± 1.5
2	CH_3_	H	H	A	20 min	3b	72.6 ± 0.9
				B	17 h		61.3 ± 1.4
3	H	CH_3_	H	A	20 min	3c	77.9 ± 0.6
				B	17 h		68.4 ± 0.7
4	H	H	CH_3_	A	20 min	3d	81.2 ± 0.5
				B	17 h		76.1 ± 1.2
5	CH_3_	H	CH_3_	A	20 min	3e	61.3 ± 1.1
				B	17 h		53.4 ± 0.7
6	H	CH_3_	CH_3_	A	20 min	3f	70.7 ± 0.9
				B	17 h		59.4 ± 1.1
7	H	H	(CH_3_)_2_CH	A	20 min	3g	85.5 ± 0.4
				B	17 h		68.6 ± 0.8
8	CH_3_	H	(CH_3_)_2_CH	A	20 min	3h	61.2 ± 1.3
				B	17 h		49.6 ± 1.4
9	H	CH_3_	(CH_3_)_2_CH	A	20 min	3i	70.3 ± 0.6
				B	17 h		61.4 ± 0.7
10	H	H	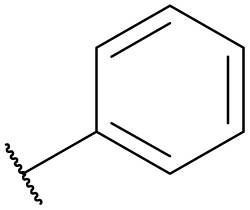	A	20 min	3j	80.3 ± 1.1
				B	17 h		70.6 ± 0.7
11	CH_3_	H	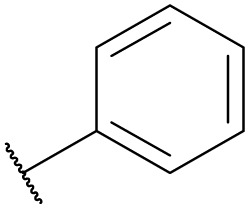	A	20 min	3k	61.2 ± 0.8
				B	17 h		50.4 ± 0.9
12	H	CH_3_	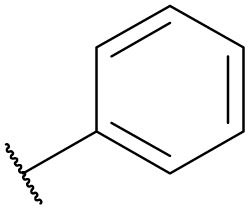	A	20 min	3l	73.3 ± 0.7
				B	17 h		66.3 ± 1.4
13	H	H	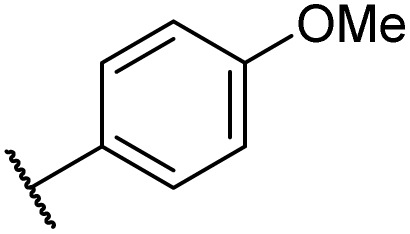	A	20 min	3m	83.5 ± 1.2
				B	17 h		71.6 ± 0.9
14	CH_3_	H	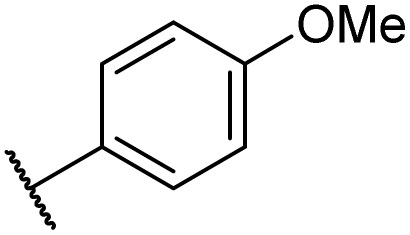	A	20 min	3n	66.5 ± 0.4
				B	17 h		54.3 ± 0.6
15	H	CH_3_	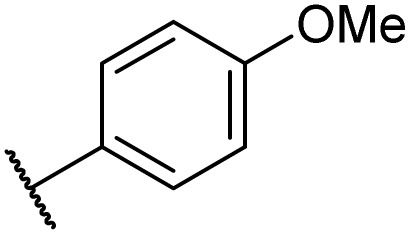	A	20 min	3o	73.5 ± 1.5
				B	17 h		67.6 ± 0.7
16	H	H	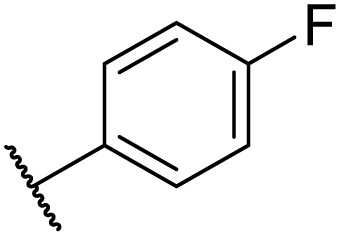	A	20 min	3p	67.7 ± 0.8
				B	17 h		51.3 ± 1.3

a General experimental conditions: Method A: continuous flow reactors, feed 1, dissolve 5 mmol of pyrazine esters derivatives in 10 mL *tert*-amyl alcohol; feed 2, dissolve 15 mmol of amines in 10 mL *tert*-amyl alcohol, flow rate 31.2 μL min^−1^, residence time 20 min, enzyme 870 mg, 45 °C. Method B: shaker reactors, add 5 mmol of pyrazine esters derivatives, 15 mmol of amines and 20 mL *tert*-amyl alcohol to a 50 mL erlenmeyer flask, enzyme 870 mg, 160 rpm, 45 °C, 17 h.

b Isolated yield. Yield: 100 × (actual obtained amount/calculated amount). The data are presented as average ± SD of triplicate experiments.

### Morpholine as platform molecules for aminolysis reactions

Morpholine is often employed in the field of medicinal chemistry for its advantageous physicochemical, biological, and metabolic properties. The morpholine ring is a versatile and readily accessible synthetic building block, it is easily introduced as an amine reagent, we explored the aminolysis reactions of pyrazine esters and morpholine in a continuous flow microreactor catalyzed by Lipozyme® TL IM from *Thermomyces lanuginosus*. As shown in Table 5, the steric hindrance of pyrazine esters also has an effect on the aminolysis reactions, and a large steric hindrance is not conducive to the reaction. Due to steric hindrance, *N*-aminomorpholine is difficult to carry out the ammonolysis reaction with pyrazine esters (*e.g.*, entry 8, trace). The corresponding compounds were synthesized parallelly, which proves the good scalability of this process.

**Table 5:** The effect of morpholine structure to the enzymatic synthesis of pyrazinamide derivatives under continuous-flow conditions^a^

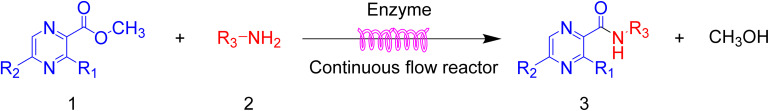							
Entry	R_1_	R_2_	R_3_	Method	Time	Product	Yield^b^ (%)
1	H	H	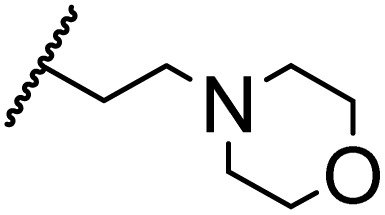	A	20 min	3q	80.6 ± 0.8
				B	17 h		71.3 ± 0.9
2	CH_3_	H	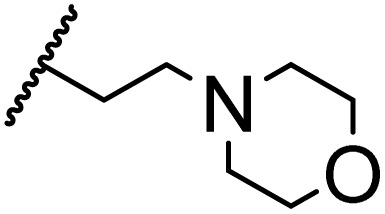	A	20 min	3r	70.9 ± 1.0
				B	17 h		58.3 ± 1.4
3	H	CH_3_	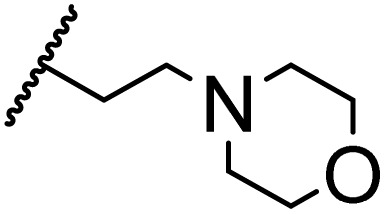	A	20 min	3s	71.3 ± 0.6
				B	17 h		63.4 ± 0.9
4	H	H	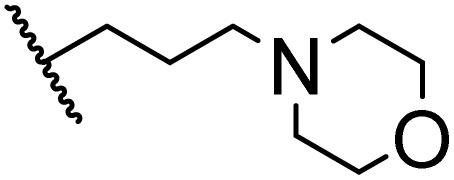	A	20 min	3t	91.6 ± 1.0
				B	17 h		84.5 ± 0.8
5	CH_3_	H	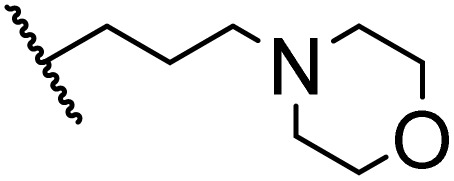	A	20 min	3u	70.3 ± 0.7
				B	17 h		60.5 ± 0.9
6	H	CH_3_	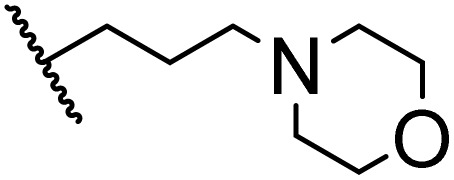	A	20 min	3v	80.5 ± 1.3
				B	17 h		71.8 ± 0.7
7	H	H	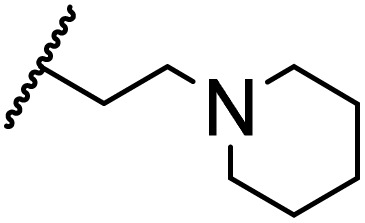	A	20 min	3w	85.8 ± 0.6
				B	17 h		74.5 ± 0.8
8	H	H	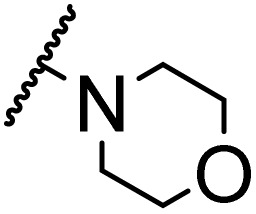	A	20 min	3x	Trace
				B	17 h		Trace
9	CH_3_	H	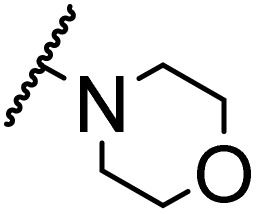	A	20 min	3y	Trace
				B	17 h		Trace
10	H	CH_3_	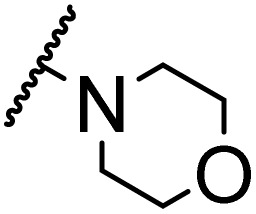	A	20 min	3z	Trace
				B	17 h		Trace

a General experimental conditions: Method A: continuous flow reactors, feed 1, dissolve 5 mmol of pyrazine esters derivatives in 10 mL *tert*-amyl alcohol; feed 2, dissolve 15 mmol of amines in 10 mL *tert*-amyl alcohol, flow rate 31.2 μL min^−1^, residence time 20 min, enzyme 870 mg, 45 °C. Method B: shaker reactors, add 5 mmol of pyrazine esters derivatives, 15 mmol of amines and 20 mL *tert*-amyl alcohol to a 50 mL erlenmeyer flask, enzyme 870 mg, 160 rpm, 45 °C, 17 h.

b Isolated yield. Yield: 100 × (actual obtained amount/calculated amount). The data are presented as average ± SD of triplicate experiments.

## Experimental section

**Fig. 7:**
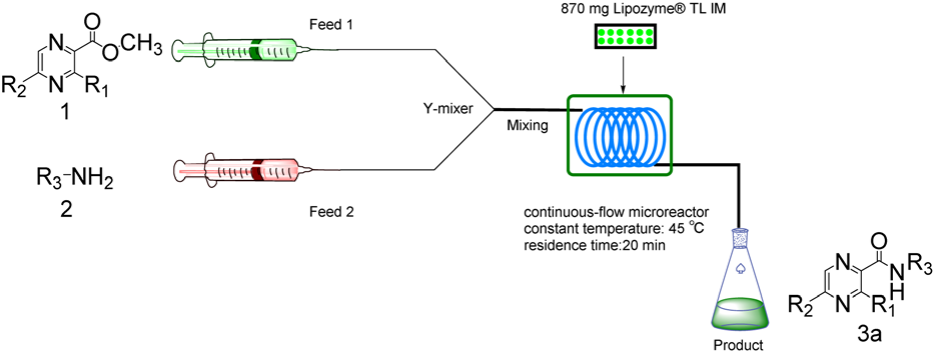
The equipment diagram for the synthesis of pyrazinamide derivatives in the continuous-flow microreactor catalysed by Lipozyme® TL IM from *Thermomyces lanuginosus*.

The equipment diagram for the synthesis of pyrazinamide derivatives in the continuous-flow microreactor is depicted in Fig. 7. The experimental setup consists of a syringe pump (Harvard Apparatus Dr 2000), two substrate injectors, a Y-mixer, a flow reactor with 100 cm × 2 mm PFA tubing and a product collector. Silica gel tubes were filled with 870 mg of Lipozyme® TL IM from *Thermomyces lanuginosus* (reactivity 250 IUN g^−1^) and then immersed in a constant temperature water bath at 45 °C. 5 mmol of pyrazine esters derivatives were dissolved in 10 mL *tert*-amyl alcohol (feed 1), and 15 mmol of amines were dissolved in 10 mL *tert*-amyl alcohol (feed 2). Feed 1 and 2 were delivered to the Y-mixer at a flow rate of 31.2 μL min^−1^ with a residence time of 20 min. The resulting stream was connected to a sample vial to collect the final mixture. The main products were separated by silica gel chromatography and were confirmed by ^1^H NMR, ^13^C NMR.

### The gram scale synthesis of pyrazinamide derivatives

**Fig. 8:**
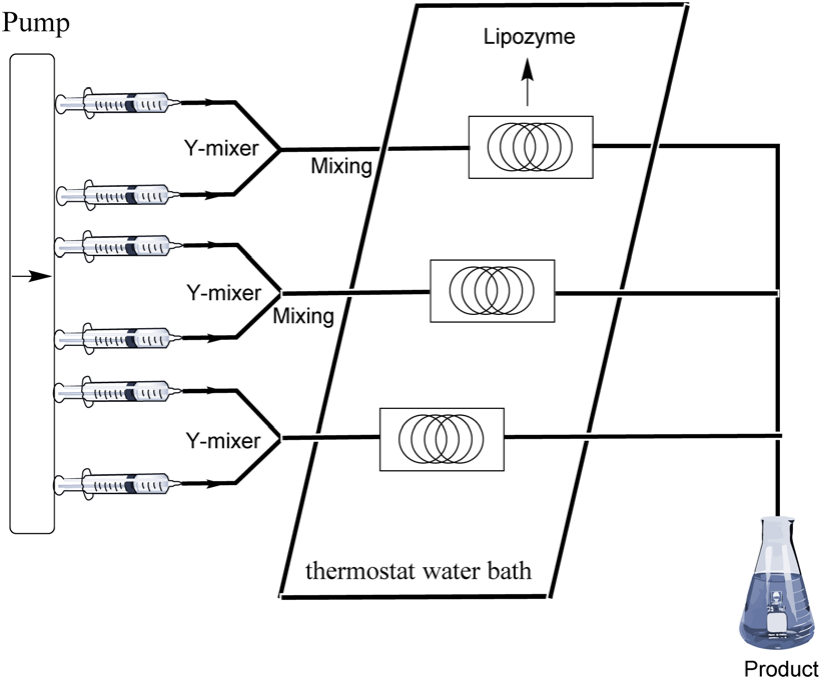
The equipment diagram for the gram scale synthesis of pyrazinamide derivatives in the continuous-flow microreactor catalysed by Lipozyme® TL IM from *Thermomyces lanuginosus*.

As shown in Fig. 8, the pump in the continuous-flow system can carry three reaction channels simultaneously. Based on the reaction conditions we explored, taking pyrazinamide (3j) as an example, a yield of 0.8562 g can be obtained from a single reaction channel, the yield of two channels in parallel is 1.7123 g, and the yield of three channels in parallel is 2.5685 g, which prove the broader scope and applicability of the methodology.

## Conclusion

In summary, we have developed a more efficient and greener synthesis method for pyrazinamide derivatives from pyrazine esters and amides (aliphatic amine, benzylamines and morpholine) in continuous-flow system catalyzed by Lipozyme® TL IM from *Thermomyces lanuginosus*, which was used for the first time. The reaction parameters such as solvent, substrate ratio, reaction temperature and reaction time/flow rate were systematically studied and gave 23 target compounds. Compared to the reported works, the significant advantages of this methodology include greener conditions (*tert*-amyl alcohol, 45 °C), shorter reaction time (20 min), a streamlined and concise route (a one-step reaction), high space–time yield (compared with shaker reactors), easy handling of the highly reusable biocatalyst and scalability applicable to various amine substrates, including biogenic amines. This approach is a complement to the existing methods for the construction of pyrazinamide derivatives, and future works will focus on further biological assays and exploring the scale-up potential of this method.

## Data availability

The authors confirm that the data supporting the findings of this study are available within its ESI.†

## Conflicts of interest

There are no conflicts to declare.

## Supplementary Material

RA-014-D4RA06761D-s001
