# Supplementary material for: Pyrazine derivative synthesis in a continuous-flow system: a green synthesis of pyrazinamide from pyrazine esters and amines catalyzed by Lipozyme® TL IM from Thermomyces lanuginosus
Source: RSC Adv. 2024 Dec 16;14(53):39560–8. doi: 10.1039/d4ra06761d (PMC11648184; doi:10.1039/d4ra06761d)
Supplement: RA-014-D4RA06761D-s001 [file RA-014-D4RA06761D-s001.pdf]

Supplementary Information (SI) for RSC Advances.
This journal is © The Royal Society of Chemistry 2024

Supporting Information for

Pyrazine derivatives Synthesis in Continuous-flow system: A Green Synthesis of Pyrazinamide from Pyrazine esters and Amines Catalyzed by Lipozyme® TL IM from *Thermomyces lanuginosus*

Ao-Ying Zhang, [#a] Zong-Hao Huang, [#a] Li-Hua Du, *[a] Hang Lin, [a] Han-Jia Xie, [a] Bing-Lin Yan, [a] Miao Miao Xue, [a] Lin Wang, [a] Wen-Xuan Shao, [a] Guo-Neng Fu [a] and Xi-Ping Luo *[b]

[a] College of Pharmaceutical Science, ZheJiang University of Technology, Hangzhou, 310014, China.

[b] Zhejiang Provincial Key Laboratory of Chemical Utilization of Forestry Biomass, Zhejiang A&F University, Hangzhou, 311300, China.

*Corresponding author. Fax: +86 18969069399. E-mail: orgdlh@zjut.edu.cn; orgdlh@gmail.com; luoxiping@zafu.edu.cn

[#] These authors contributed equally to this work and should be considered co-first authors.

Materials

All chemicals in this study were obtained from commercial sources and did not require further purification. Lipozyme® TL IM (immobilized *Thermomyces lanuginosus*) was purchased from Novo Nordisk (Copenhagen, Denmark). Pyrazine-2-carboxylate, methyl 3-methylpyrazine-2-carboxylate, methyl 5-mathylpyrazine-2-carboxylate and methylamine were purchased from Aladdin (Shanghai, China). Ethylamine, isobutylamine, benzylamine, 4-methoxybenzylamine and 4-fluorobenzylamine were purchased from Energy Chemical (Shanghai, China). N-aminomorpholine, N-(2-aminoethyl)morpholine, N-(3-aminopropyl)morpholine were purchased from Macklin (Shanghai, China). Harvard Instrument PHD 2000 syringe pump was purchased from Harvard University (Holliston, Massachusetts, USA). The flow reactor and Y-mixer were purchased from Beijing Haigui Medical Engineering Design Co., Ltd (Beijing China).

Purification of the product

When the conversion of the pyrazinamide derivative reaches a maximum (determined by TLC), the reaction is terminated by filtering the enzyme, and the *tert*-amyl alcohol solvent is rotary evaporated under reduced pressure. The product is separated by silica gel chromatography (mobile phase petroleum ether / ethyl acetate, 6/1 to 1/1). Purification

was monitored by TLC. The graded fractions containing the major product were combined, the solvent evaporated and the residue analyzed by ^{1}H NMR, ^{13}C NMR.

Experimental setup

A continuous-flow microreactor equipment diagram which was used for synthesis of pyrazinamide derivatives from pyrazine esters and amines catalyzed by Lipozyme® TL IM is described in Figure S1. The experimental setup consists of a syringe pump, two substrate injectors, Y-shaped mixers (φ= 1.8 mm) and a product collector. Syringe pumps (Harvard apparatus PHD 2000) were used to introduce separate feed streams to the flow reactor with 100cm × 2 mm PFA tubing. Silica gel tubes were filled with Lipozyme® TL IM and immersed in a constant temperature water bath to control the temperature. A total of 5 mmol of pyrazine esters were dissolved in 10 mL of *tert*-amyl alcohol (feed 1), and 15 mmol of amines were dissolved in 10 mL of *tert*-amyl alcohol (feed 2). Feeds 1 and 2 were placed in separate 10 mL feeders and mixed at a flow rate of 31.2 μL min^{-1} in a Y-mixer at 45 °C. The resulting stream (31.2 μL min^{-1}) was connected to a sample vial for collection of the final mixture.

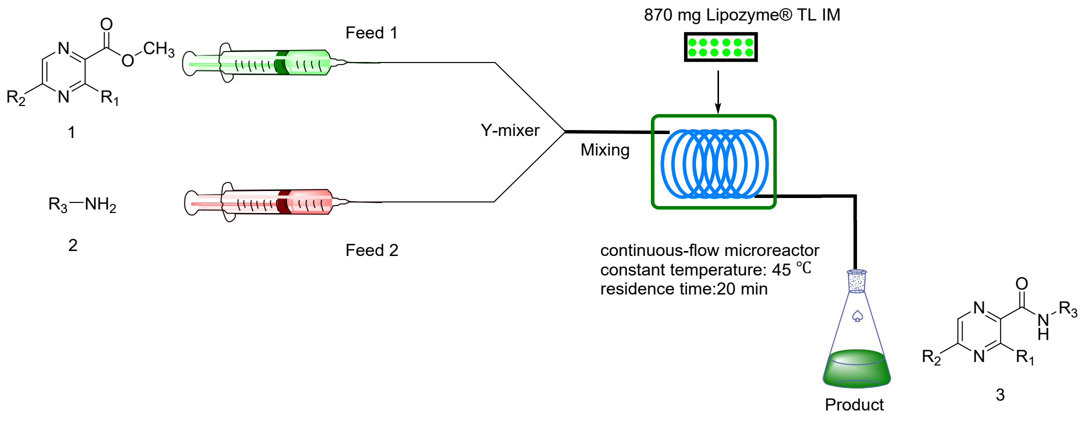

Figure S1. The equipment diagram for the synthesis of pyrazinamide derivatives in the continuous-flow microreactor catalyzed by Lipozyme® TL IM.

General Procedure for the synthesis of pyrazinamide derivatives from pyrazine esters and amines catalyzed by Lipozyme® TL IM in Continuous-Flow Microreactors

Method A: 5.0 mmol of pyrazine esters was dissolved in 10 mL *tert*-amyl alcohol (feed A, ~0.5 M) and 15.0 mmol amine were dissolved in 10 mL *tert*-amyl alcohol (feed B; ~1.0 M). Lipozyme® TL IM (0.87 g) were filled in PFA reactor coil (inner

diameter ID= 2.0 mm, length = 100cm.). Streams A and B were mixed together at a flow rate of 15.6 μL min^{-1} in a Y-mixer at 45 °C and the resulting stream (31.2 μL min^{-1}) was connected to a sample vial which was used to collect the final mixture. The final mixture was then evaporated, and the residue was submitted to column chromatography on silica gel (200-300 mesh). The crude product was purified by silica gel column chromatography with a petroleum ether / ethyl acetate gradient from 6:1 to 1:1. The purification was monitored by TLC. The fractions containing the main products were pooled, the solvent evaporated, and the residue analyzed by ^{1}H NMR, and ^{13}C NMR.

General Procedure for the synthesis of pyrazinamide derivatives from pyrazine esters and amines catalyzed by Lipozyme® TL IM under Shaker Conditions

Method B: pyrazine esters (5.0 mmol) and amine (15.0 mmol) were added to 20 mL *tert*-amyl alcohol. The biocatalyst Lipozyme® TL IM (0.87 g) was then added and the suspension maintained at 45 °C for 17 h under Shaker Conditions (200 r·min^{-1}). The mixture was cooled and filtered. Then evaporated under reduced pressure and the residue was submitted to column chromatography on silica gel (200-300 mesh). The crude product was purified by silica gel column chromatography with a petroleum ether /ethyl acetate gradient from 6:1 to 1:1. The purification was monitored by TLC. The fractions containing the main products were pooled, the solvent evaporated, and the residue analyzed by ^{1}H NMR, and ^{13}C NMR.

Experimental data of products

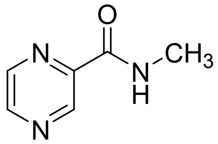

N-methylpyrazine-2-carboxamide (3a). White powder, 86.4% yield, m.p. 105°C.[1] ^{1}H NMR (400 MHz, Chloroform-*d*) δ 9.38 (d, *J* = 1.4 Hz, 1H), 8.72 (d, *J* = 2.5 Hz, 1H), 8.49 (dd, *J* = 2.3, 1.6 Hz, 1H), 7.82 (br s, 1H, -NH), 3.03 (d, *J* = 5.1 Hz, 3H). ^{13}C NMR (101 MHz, Chloroform-*d*) δ 163.60, 147.21, 144.50, 144.28, 142.55, 26.15.

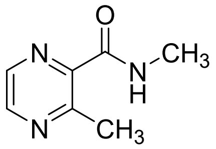

N,3-dimethylpyrazine-2-carboxamide (3b). White powder, 72.6% yield, m.p. 88°C. ^{1}H NMR (400 MHz, Chloroform-*d*) δ 8.55 (d, *J* = 2.4 Hz, 1H), 8.31 (d, *J* = 2.4 Hz, 1H), 7.90 (br s, 1H, -NH), 3.00 – 2.93 (m, 6H). ^{13}C NMR (101 MHz, Chloroform-*d*) δ 165.19, 155.28, 145.71, 142.80, 139.89, 26.10, 23.65.

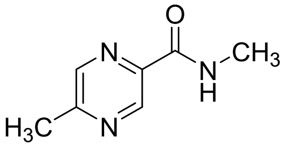

N,5-dimethylpyrazine-2-carboxamide (3c). White powder, 77.9% yield, m.p. 130°C. ^{1}H NMR (400 MHz, Chloroform-*d*) δ 9.24 (d, *J* = 1.9 Hz, 1H), 8.35 (d, *J* = 1.5 Hz, 1H), 7.78 (br s, 1H, -NH), 3.02 (s, 3H), 2.62 (d, *J* = 3.8 Hz, 3H). ^{13}C NMR (101 MHz, Chloroform-*d*) δ 163.91, 156.90, 143.14, 142.29, 141.83, 26.03, 21.76.

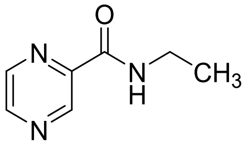

N-ethylpyrazine-2-carboxamide (3d). White solid, 81.2% yield, m.p. 67°C.[2] ^{1}H NMR (400 MHz, Chloroform-*d*) δ 9.39 (d, *J* = 1.5 Hz, 1H), 8.73 (d, *J* = 2.5 Hz, 1H), 8.51 (dd, *J* = 2.5, 1.6 Hz, 1H), 7.82 (br s, 1H, -NH), 3.52 (qd, *J* = 7.3, 5.8 Hz, 2H), 1.26 (t, *J* = 7.3 Hz, 3H). ^{13}C NMR (101 MHz, Chloroform-*d*) δ 162.79, 147.14, 144.60, 144.34, 142.50, 34.34, 14.79.

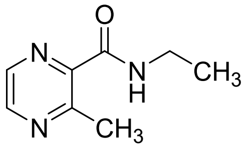

N-ethyl-3-methylpyrazine-2-carboxamide (3e). Yellowish oily liquid, 61.3% yield. ^{1}H NMR (400 MHz, Chloroform-*d*) δ 8.53 (d, *J* = 2.4 Hz, 1H), 8.30 (d, *J* = 2.4 Hz, 1H), 7.88 (br s, 1H, -NH), 3.49 – 3.37 (m, 2H), 2.93 (s, 3H), 1.21 (t, *J* = 7.3 Hz, 3H). ^{13}C NMR (101 MHz, Chloroform-*d*) δ 164.43, 155.28, 145.64, 142.89, 139.88, 34.27, 23.64, 14.74.

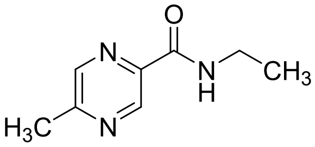

N-ethyl-5-methylpyrazine-2-carboxamide (3f). White crystal, 70.7% yield, m.p. 86°C. ^{1}H NMR (400 MHz, Chloroform-*d*) δ 9.21 (d, *J* = 1.8 Hz, 1H), 8.32 (d, *J* = 1.8 Hz, 1H), 7.73 (br s, 1H, -NH), 3.53 – 3.40 (m, 2H), 2.59 (s, 3H), 1.22 (t, *J* = 4.1 Hz, 3H). ^{13}C NMR (101 MHz, Chloroform-*d*) δ 163.10, 156.84, 143.23, 142.22, 141.91, 34.22, 21.76, 14.81.

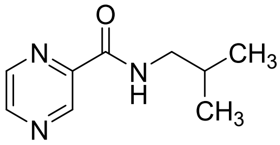

N-isobutylpyrazine-2-carboxamide (3g). White solid, 85.5% yield, m.p. 61°C. ^{1}H NMR (400 MHz, Chloroform-*d*) δ 9.41 (d, *J* = 1.5 Hz, 1H), 8.74 (d, *J* = 2.5 Hz, 1H), 8.52 (dd, *J* = 2.6, 1.5 Hz, 1H), 7.90 (br s, 1H, -NH), 3.32 (t, J = 6.1 Hz, 2H), 1.92 (hept, *J* = 6.7 Hz, 1H), 0.99 (d, *J* = 6.7 Hz, 6H). ^{13}C NMR (101 MHz, Chloroform-*d*) δ 162.93, 147.14, 144.61, 144.42, 142.48, 46.74, 28.67, 20.15.

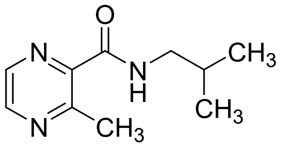

N-isobutyl-3-methylpyrazine-2-carboxamide (3h). Yellowish oily liquid, 61.2% yield. ^{1}H NMR (400 MHz, Chloroform-*d*) δ 8.58 (d, *J* = 2.4 Hz, 1H), 8.35 (d, *J* = 2.5 Hz, 1H), 8.01 (br s, 1H, -NH), 3.26 (t, *J* = 6.8 Hz, 2H), 2.97 (s, 3H), 1.90 (hept, *J* = 7.9 Hz, 1H), 0.97 (d, *J* = 6.8 Hz, 6H). ^{13}C NMR (101 MHz, Chloroform-*d*) δ 164.55, 155.38, 145.66, 142.93, 139.90, 46.77, 28.63, 23.69, 20.19.

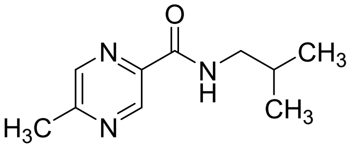

N-isobutyl-5-methylpyrazine-2-carboxamide (3i). White powder, 70.3% yield, m.p. 115°C. ^{1}H NMR (400 MHz, Chloroform-*d*) δ 9.26 (s, 1H), 8.37 (s, 1H), 7.84 (br s, 1H, -NH), 3.30 (t, *J* = 6.6 Hz, 2H), 2.64 (s, 3H), 1.91 (hept, *J* = 6.7 Hz, 1H), 0.97 (d, *J* = 1.3 Hz, 6H). ^{13}C NMR (101 MHz, Chloroform-*d*) δ 163.23, 156.85, 143.30, 142.24, 141.92, 46.66, 28.68, 21.78, 20.15.

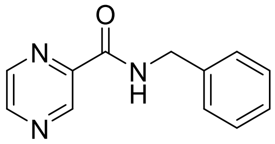

N-benzylpyrazine-2-carboxamide (3j). White solid, 80.3% yield, m.p. 117°C.[3] ^{1}H NMR (400 MHz, Chloroform-*d*) δ 9.47 (s, 1H), 8.76 (d, *J* = 2.7 Hz, 1H), 8.52 (d, *J* = 2.7 Hz, 1H), 8.17 (br s, 1H, -NH), 7.41 – 7.36 (m, 4H), 7.36 – 7.28 (m, 1H), 4.70 (d, *J* = 6.0 Hz, 2H). ^{13}C NMR (101 MHz, Chloroform-*d*) δ 162.92, 147.35, 144.55, 144.43, 142.56, 137.76, 128.82, 127.90, 127.71, 43.52.

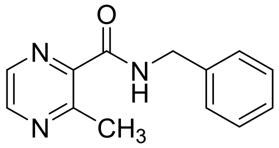

N-benzyl-3-methylpyrazine-2-carboxamide (3k). Yellowish oily liquid, 61.2% yield, m.p. 78°C. ^{1}H NMR (400 MHz, Chloroform-*d*) δ 8.58 (d, *J* = 2.4 Hz, 1H), 8.33 (d, *J* = 2.4 Hz, 1H), 8.27 (br s, 1H, -NH), 7.39 – 7.30 (m, 4H), 7.34 – 7.24 (m, 1H), 4.63 (d, *J* = 6.0 Hz, 2H), 3.02 (s, 3H). ^{13}C NMR (101 MHz, Chloroform-*d*) δ 164.44, 155.58, 145.88, 142.65, 139.95, 138.10, 128.77, 127.84, 127.57, 43.49, 23.75.

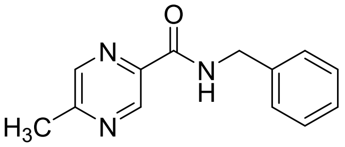

N-benzyl-5-methylpyrazine-2-carboxamide (3l). White powder, 73.3% yield, m.p. 108°C. ^{1}H NMR (400 MHz, Chloroform-*d*) δ 9.30 (s, 1H), 8.34 (s, 1H), 8.09 (br s, 1H, -NH), 7.37 –

7.31 (m, 4H), 7.33 – 7.25 (m, 1H), 4.66 (d, *J* = 6.0 Hz, 2H), 2.63 (s, 3H). ^{13}C NMR (101 MHz, Chloroform-*d*) δ 163.24, 157.13, 143.46, 142.32, 141.74, 137.92, 128.78, 127.88, 127.63, 43.43, 21.85.

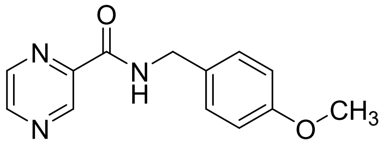

N-(4-methoxybenzyl)pyrazine-2-carboxamide (3m). White solid, 83.5% yield, m.p. 134°C. ^{1}H NMR (400 MHz, Chloroform-*d*) δ 9.44 (s, 1H), 8.74 (d, *J* = 2.4 Hz, 1H), 8.49 (d, *J* = 2.7 Hz, 1H), 8.10 (br s, 1H, -NH), 7.31 (d, *J* = 8.3 Hz, 1H), 6.89 (d, *J* = 8.5 Hz, 1H), 4.62 (d, *J* = 5.9 Hz, 2H), 3.80 (s, 3H). ^{13}C NMR (101 MHz, Chloroform-*d*) δ 162.80, 159.16, 147.27, 144.50, 142.56, 129.84, 129.29, 114.16, 55.32, 43.01.

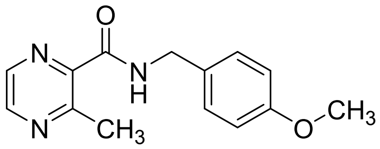

N-(4-methoxybenzyl)-3-methylpyrazine-2-carboxamide (3n). White powder, 66.5% yield, m.p. 93°C. ^{1}H NMR (400 MHz, Chloroform-*d*) δ 8.57 (d, *J* = 2.4 Hz, 1H), 8.32 (d, *J* = 2.4 Hz, 1H), 8.19 (br s, 1H, -NH), 7.32 – 7.25 (d, *J* = 8.6 Hz, 2H), 6.87 (d, *J* = 8.7 Hz, 2H), 4.56 (d, *J* = 5.9 Hz, 2H), 3.79 (s, 3H), 3.01 (s, 3H). ^{13}C NMR (101 MHz, Chloroform-*d*) δ 164.33, 159.09, 155.51, 145.78, 142.73, 139.94, 130.17, 129.22, 114.14, 55.31, 42.98, 23.72.

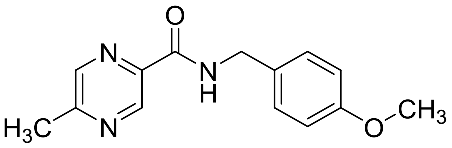

N-(4-methoxybenzyl)-5-methylpyrazine-2-carboxamide (3o). White powder, 73.5% yield, m.p. 117°C. ^{1}H NMR (400 MHz, Chloroform-*d*) δ 9.28 (s, 1H), 8.33 (s, 1H), 8.00 (br s, 1H, -NH), 7.28 (d, *J* = 8.4 Hz, 2H), 6.86 (d, *J* = 8.3 Hz, 2H), 4.58 (d, *J* = 5.9 Hz, 2H), 3.78 (s, 3H), 2.63 (s, 3H). ^{13}C NMR (101 MHz, Chloroform-*d*) δ 163.11, 159.12, 157.05, 143.41, 142.31, 141.81, 129.99, 129.29, 114.14, 55.31, 42.93, 21.82.

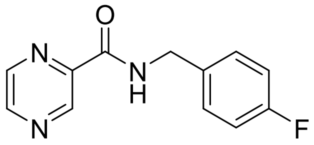

N-(4-fluorobenzyl)pyrazine-2-carboxamide (3p). White solid, 67.7% yield, m.p. 134°C.[4] ^{1}H NMR (400 MHz, Chloroform-*d*) δ 9.42 (d, *J* = 1.6 Hz, 1H), 8.73 (d, *J* = 2.4 Hz, 1H), 8.49 (dd, *J* = 2.0, 1.1 Hz, 1H), 8.14 (br s, 1H, -NH), 7.32 (dd, *J* = 8.4, 5.4 Hz, 2H), 7.01 (t, *J* = 8.6 Hz, 2H), 4.63 (d, *J* = 6.1 Hz, 2H). ^{13}C NMR (101 MHz, Chloroform-*d*) δ 163.50, 162.94(d, 1 Jc–f = 243.67 Hz), 147.40, 144.53, 144.33, 142.57, 133.64(d, 4 Jc–f = 3.40 Hz), 129.62(d, 3 Jc–f = 8.20 Hz), 115.75(d, 2 Jc–f = 21.42 Hz), 42.77. ^{19}F NMR (377 MHz, Chloroform-*d*) δ -114.51 – -114.70 (m).

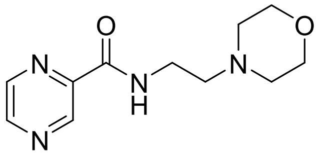

N-(2-morpholinoethyl)pyrazine-2-carboxamide (3q). White crystal, 80.6% yield, m.p. 91°C. ^{1}H NMR (400 MHz, Chloroform-*d*) δ 9.39 (d, *J* = 1.5 Hz, 1H), 8.74 (d, *J* = 2.4 Hz, 1H), 8.55 (dd, *J* = 2.5, 1.5 Hz, 1H), 8.19 (br s, 1H, -NH), 3.74 (t, *J* = 4.0 Hz, 4H), 3.60 (q, *J* = 5.9 Hz, 2H), 2.62 (t, *J* = 6.1 Hz, 2H), 2.52 (t, *J* = 4.7 Hz, 4H). ^{13}C NMR (101 MHz, Chloroform-*d*) δ 163.05, 147.18, 144.62, 144.38, 142.66, 66.95, 57.05, 53.42, 35.84.

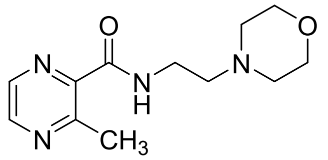

3-methyl-N-(2-morpholinoethyl)pyrazine-2-carboxamide (3r). Yellow oily liquid, 70.9% yield. ^{1}H NMR (400 MHz, Chloroform-*d*) δ 8.52 (d, *J* = 7.4 Hz, 1H), 8.31 (d, *J* = 10.9 Hz, 1H), 8.17 (br s, 1H, -NH), 3.66 (t, *J* = 4.1 Hz, 4H), 3.48 (q, *J* = 5.6 Hz, 2H), 2.90 (s, 3H), 2.53 (t, *J* = 4.1 Hz, 2H), 2.44 (t, J = 6.0 Hz, 4H). ^{13}C NMR (101 MHz, Chloroform-*d*) δ 164.66, 155.17, 145.63, 142.96, 140.04, 66.87, 57.13, 53.38, 35.86, 23.59.

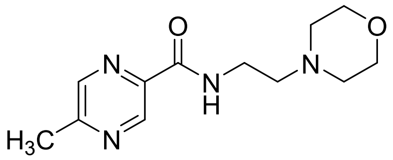

5-methyl-N-(2-morpholinoethyl)pyrazine-2-carboxamide (3s). Yellow crystal, 71.3% yield, m.p. 80°C. ^{1}H NMR (400 MHz, Chloroform-*d*) δ 9.22 (s, 1H), 8.37 (s, 1H), 8.08 (br s, 1H, -NH), 3.70 (t, *J* = 4.5 Hz, 4H), 3.55 (q, *J* = 6.0 Hz, 2H), 2.61 (s, 3H), 2.58 (t, *J* = 4.1 Hz, 2H), 2.48 (t, *J* = 6.0 Hz, 4H). ^{13}C NMR (101 MHz, Chloroform-*d*) δ 163.35, 156.89, 143.28, 142.40, 141.93, 66.97, 57.11, 53.42, 35.80, 21.81.

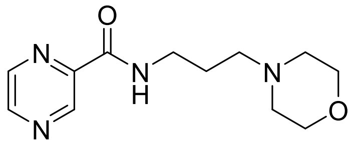

N-(3-morpholinopropyl)pyrazine-2-carboxamide (3t). Yellowish oily liquid, 91.6% yield. ^{1}H NMR (400 MHz, Chloroform-*d*) δ 9.39 (d, *J* = 1.5 Hz, 1H), 9.06 (br s, 1H, -NH), 8.73 (d, *J* = 2.4 Hz, 1H), 8.54 (dd, *J* = 7.7, 4.0 Hz,1H), 3.79 (t, *J* = 4.7 Hz, 4H), 3.59 (q, *J* = 5.9 Hz, 2H), 2.51 (dt, *J* = 15.9, 5.4 Hz, 6H), 1.80 (p, *J* = 6.2 Hz, 2H). ^{13}C NMR (101 MHz, Chloroform-*d*) δ 163.06, 147.08, 144.86, 144.44, 142.52, 66.84, 58.14, 53.83, 39.59, 24.84.

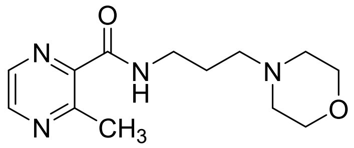

3-methyl-N-(3-morpholinopropyl)pyrazine-2-carboxamide (3u). Yellow oily liquid, 70.3% yield. ^{1}H NMR (400 MHz, Chloroform-*d*) δ 8.91 (br s, 1H, -NH), 8.56 (d, *J* = 2.4 Hz, 1H), 8.35 (d, J = 2.4 Hz, 1H), 3.73 (t, *J* = 4.0 Hz, 4H), 3.51 (q, *J* = 6.0 Hz, 2H), 2.95 (s, 3H), 2.49 (t, *J* = 4.1 Hz, 4H), 2.45 (t, *J* = 4.2 Hz, 2H), 1.77 (p, *J* = 6.3 Hz, 2H). ^{13}C NMR (101 MHz, Chloroform-*d*) δ 164.68, 155.27, 145.58, 143.21, 139.90, 66.85, 57.96, 53.78, 39.30, 25.07, 23.67.

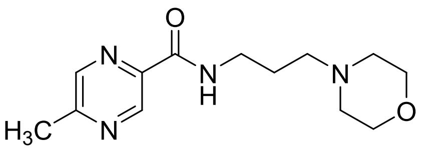

5-methyl-N-(3-morpholinopropyl)pyrazine-2-carboxamide (3v). Yellow crystal, 80.5% yield, m.p. 87°C. ^{1}H NMR (400 MHz, Chloroform-*d*) δ 9.24 (s, 1H), 8.98 (br s, 1H, -NH), 8.39 (s, 1H), 3.78 (t, *J* = 4.7 Hz, 4H), 3.57 (q, *J* = 5.8 Hz, 2H), 2.63 (s, 3H), 2.51 (dt, *J* = 16.0, 5.6 Hz, 6H), 1.78 (p, *J* = 6.3 Hz, 2H). ^{13}C NMR (101 MHz, Chloroform-*d*) δ 163.33, 156.77, 143.34, 142.24, 142.17, 66.84, 58.17, 53.83, 39.48, 24.91, 21.76.

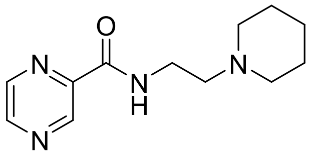

N-(2-(piperidin-1-yl)ethyl)pyrazine-2-carboxamide (3w). White crystal, 85.8% yield, m.p. 88°C. ^{1}H NMR (400 MHz, Chloroform-*d*) δ 9.39 (d, *J* = 1.5 Hz, 1H), 8.73 (d, *J* = 2.5 Hz, 1H), 8.55 (dd, *J* = 7.6, 2.0 Hz, 1H), 8.23 (br s, 1H, -NH), 3.58 (q, *J* = 6.0 Hz, 2H), 2.57 (t, *J* = 6.3 Hz, 2H), 2.45 (t, *J* = 5.2 Hz, 4H), 1.60 (p, *J* = 5.6 Hz, 4H), 1.50 – 1.40 (m, 2H). ^{13}C NMR (101 MHz, Chloroform-*d*) δ 163.01, 147.04, 144.80, 144.37, 142.66, 57.22, 54.40, 36.31, 25.97, 24.35.

3a

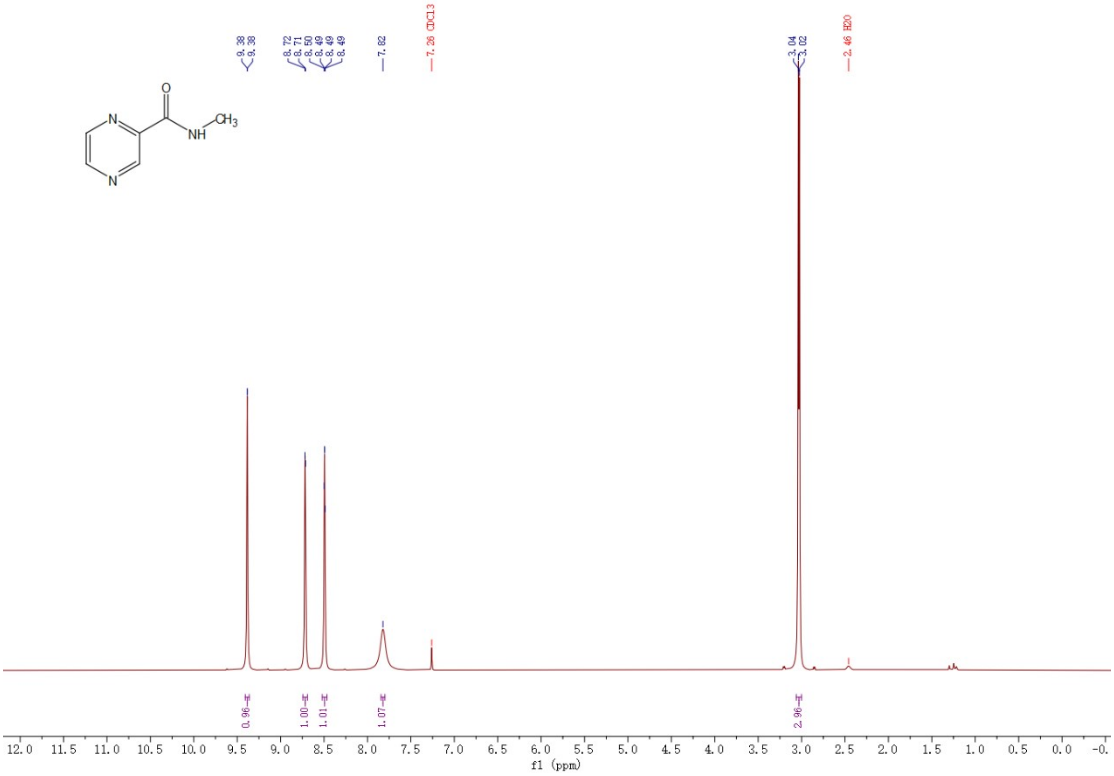
9.38
9.38
8.72
8.71
8.50
8.49
8.49
8.49
7.82
7.26 CDCl3
3.04
3.02
2.46 H2O
O
N
NH
CH3
N
0.96
1.00
1.01
1.07
2.96
12.0
11.5
11.0
10.5
10.0
9.5
9.0
8.5
8.0
7.5
7.0
6.5
6.0
5.5
5.0
4.5
4.0
3.5
3.0
2.5
2.0
1.5
1.0
0.5
0.0
f1 (ppm)

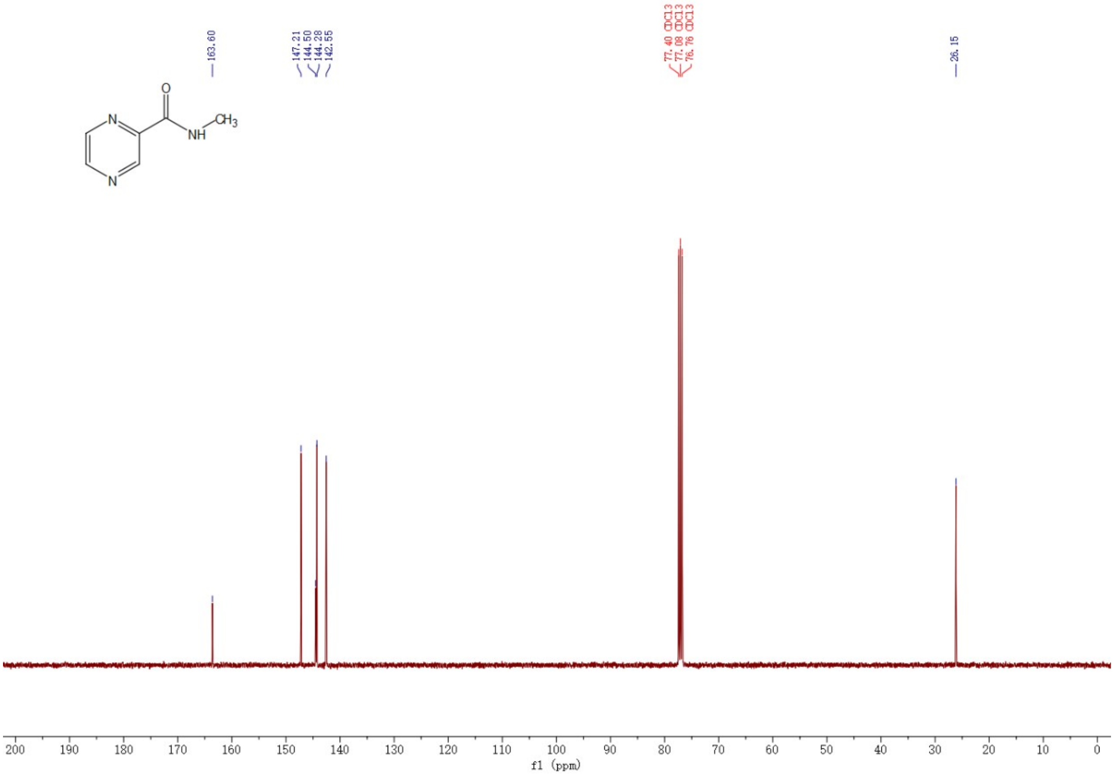
163.60
147.21
144.50
144.28
142.55
77.40 CDCl3
77.08 CDCl3
76.76 CDCl3
26.15
O
N
NH
CH3
N
200
190
180
170
160
150
140
130
120
110
100
90
80
70
60
50
40
30
20
10
0
f1 (ppm)

3b

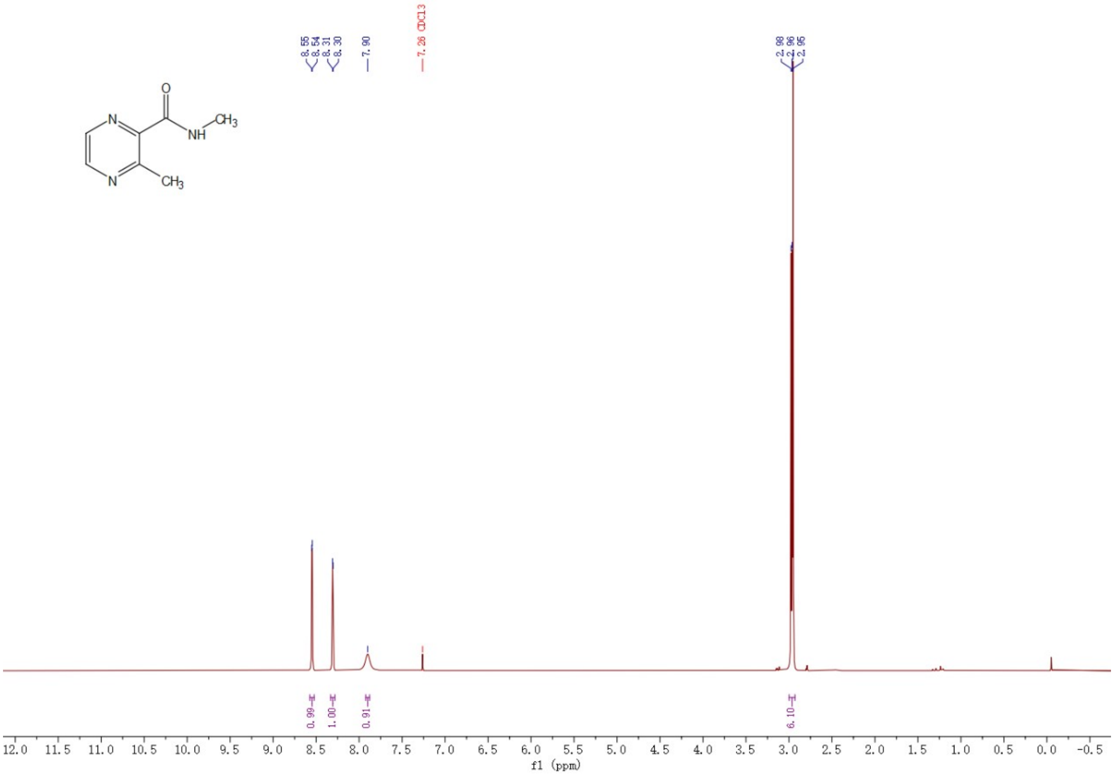
8.55
8.54
8.31
8.30
7.90
7.26 CDCl3
2.98
2.96
2.95
0.99
1.00
0.91
6.10
f1 (ppm)

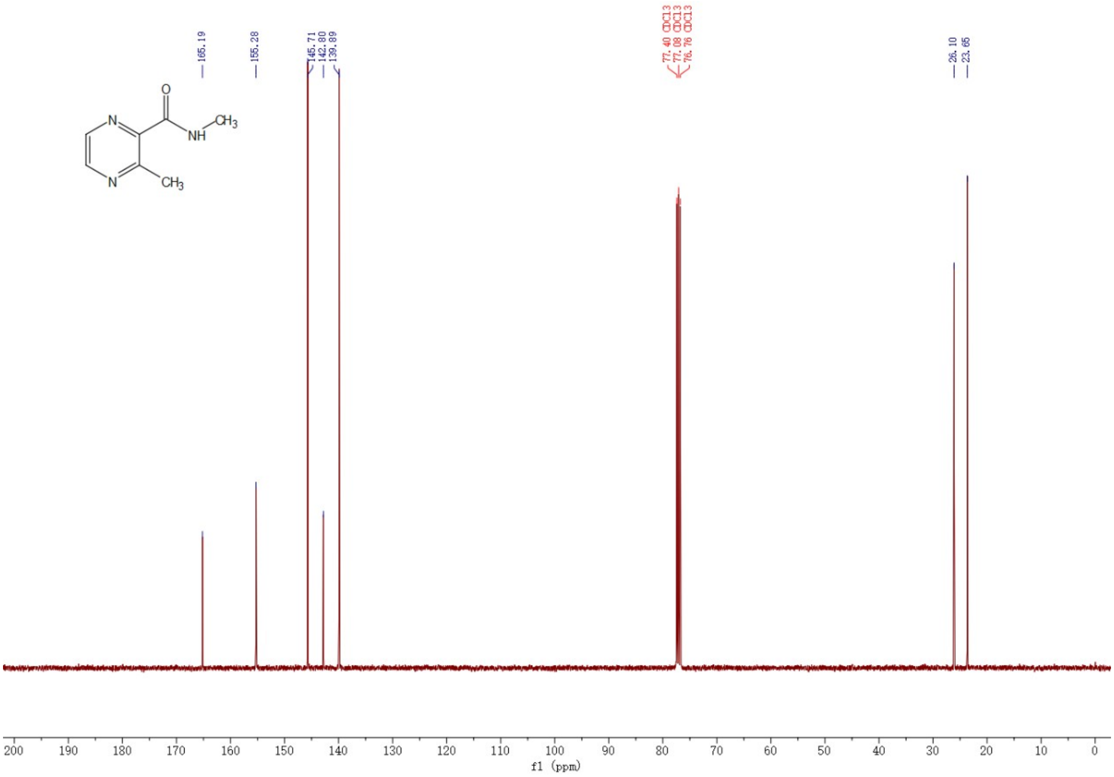
165.19
155.28
145.71
142.80
139.89
77.40 CDCl3
77.08 CDCl3
76.76 CDCl3
26.10
23.65
f1 (ppm)

3c

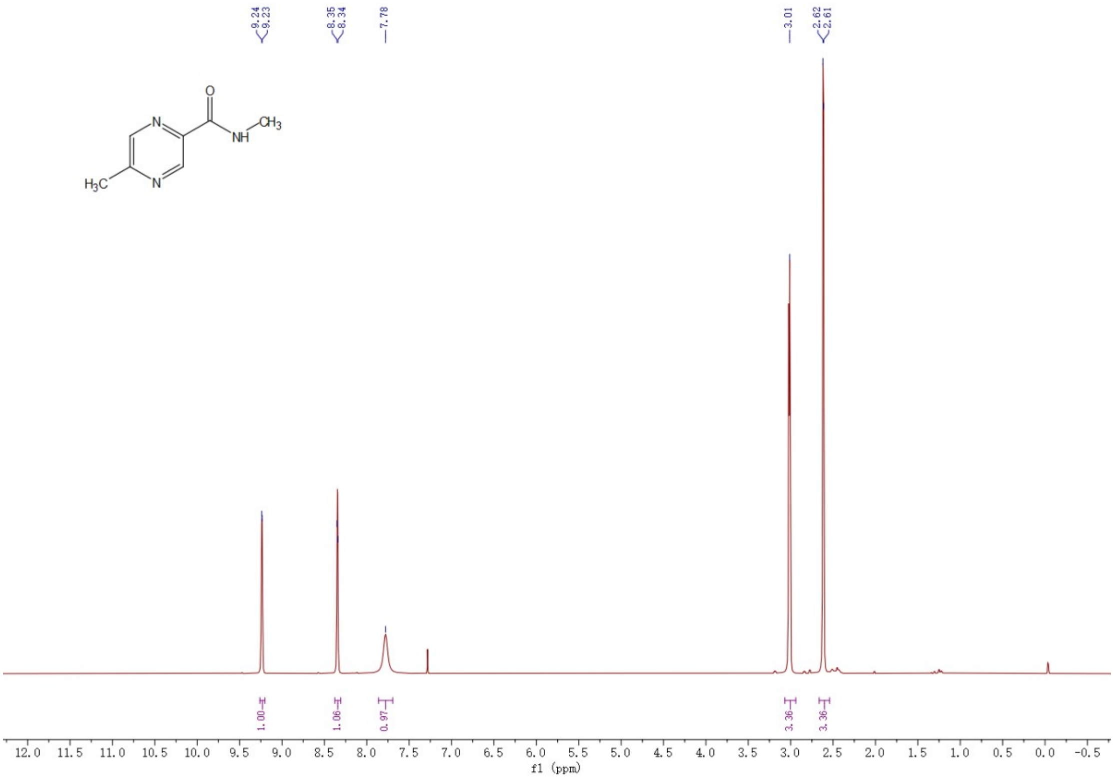

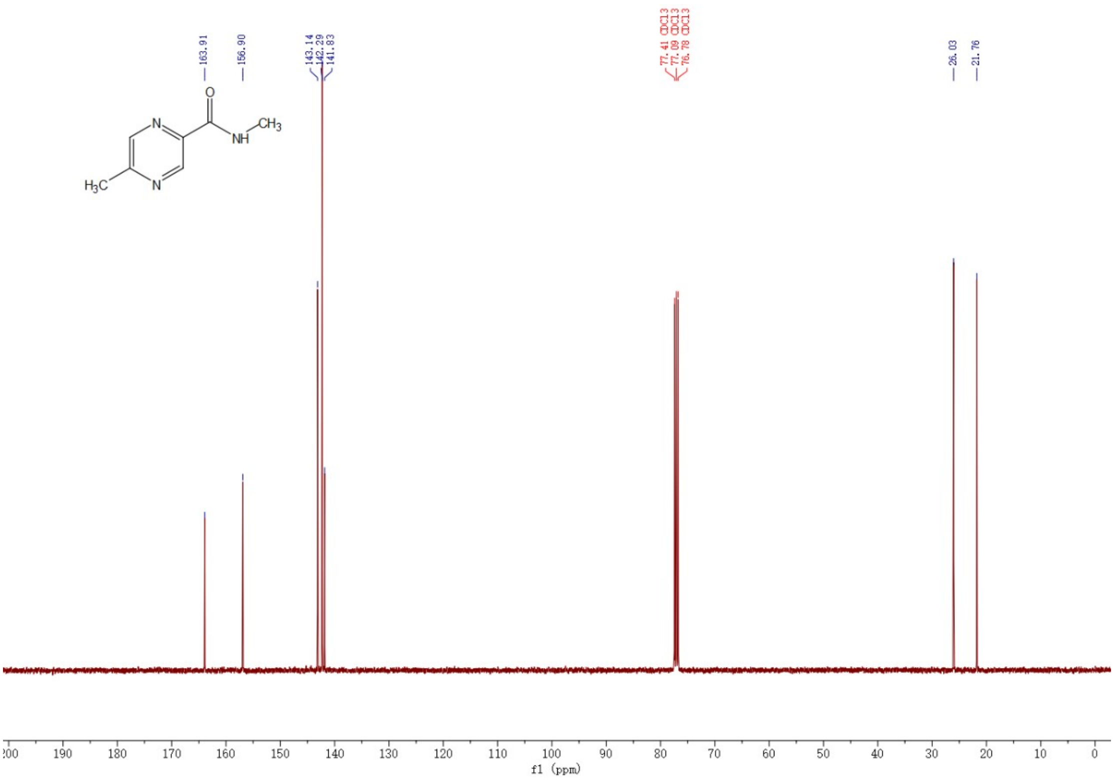

3d

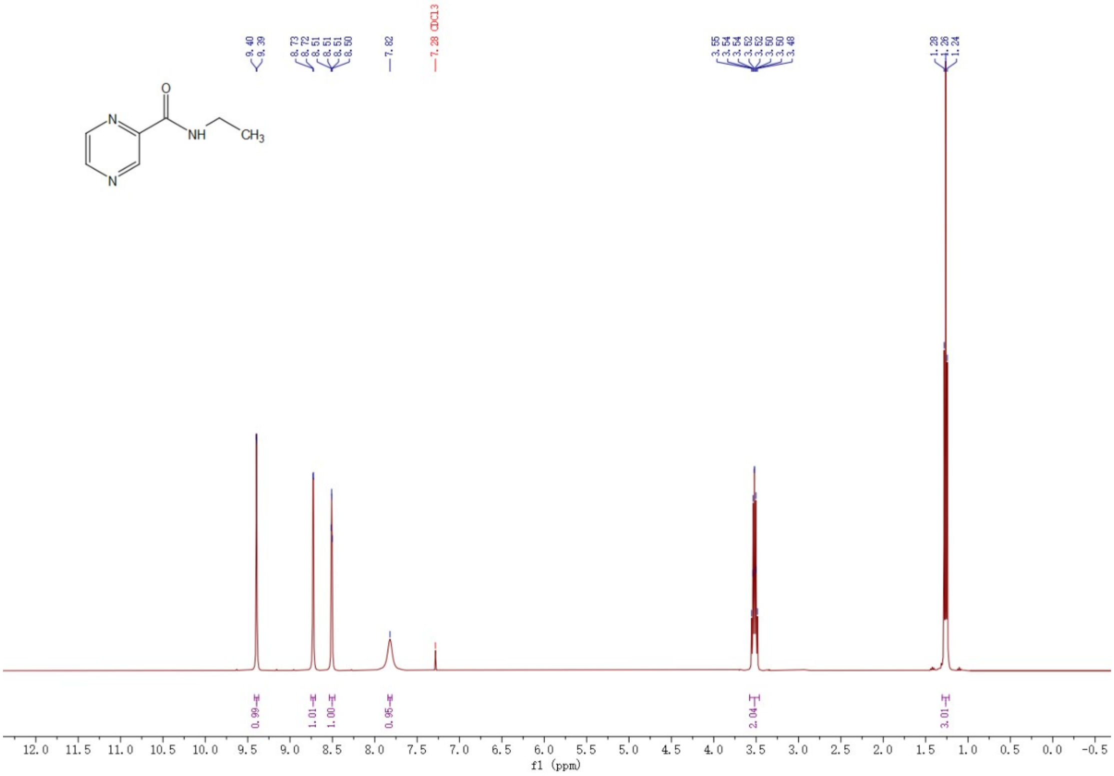

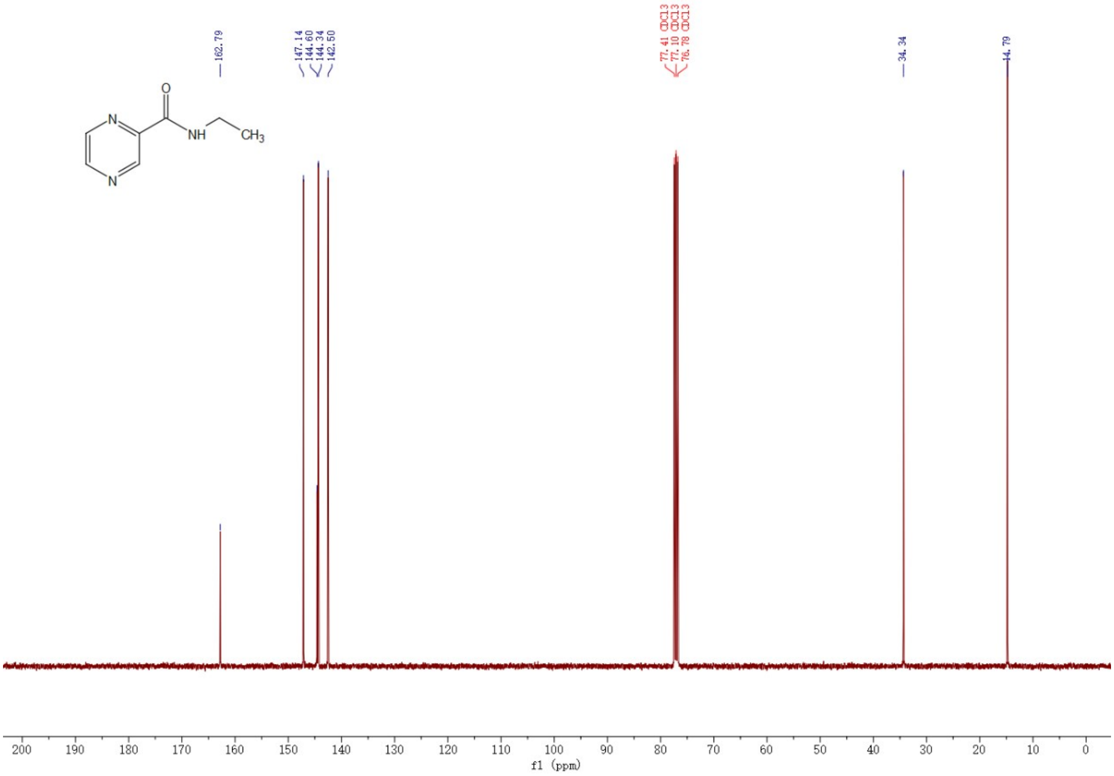

3e

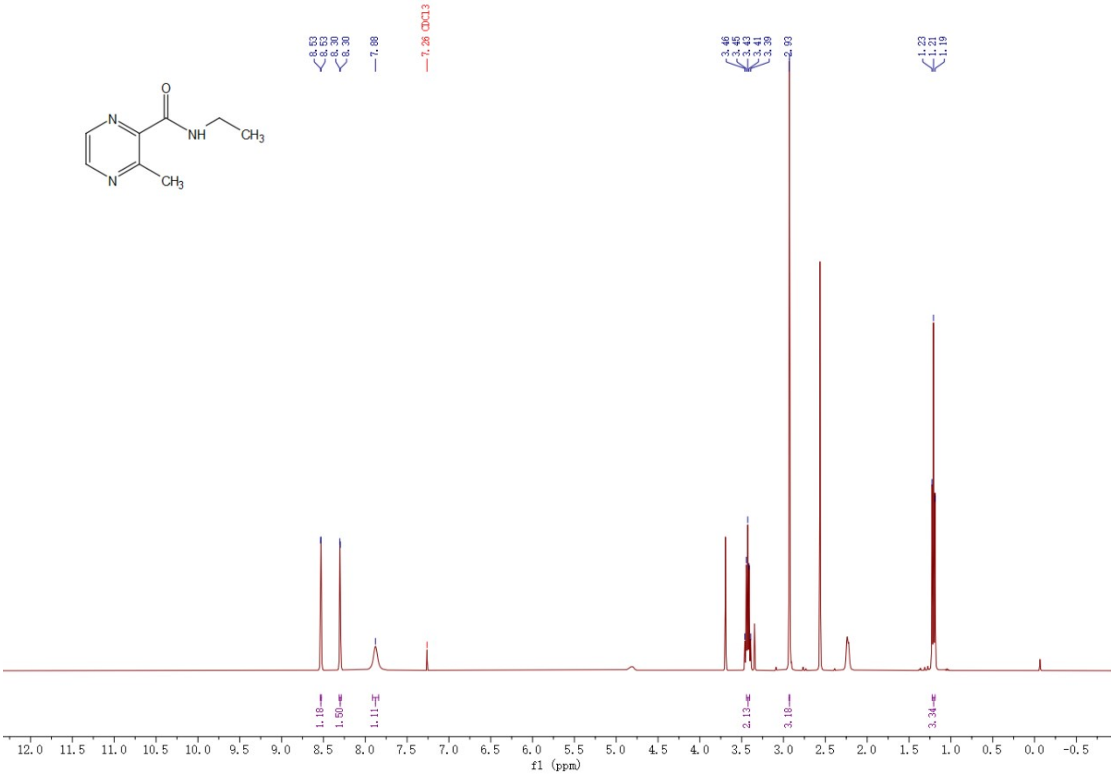

8.53
8.53
8.30
8.30
7.88
7.26 CDCl3
3.46
3.45
3.43
3.41
3.39
2.93
1.23
1.21
1.19
1.18
1.50
1.11
2.13
3.18
3.34
12.0 11.5 11.0 10.5 10.0 9.5 9.0 8.5 8.0 7.5 7.0 6.5 6.0 5.5 5.0 4.5 4.0 3.5 3.0 2.5 2.0 1.5 1.0 0.5 0.0 -0.5
f1 (ppm)

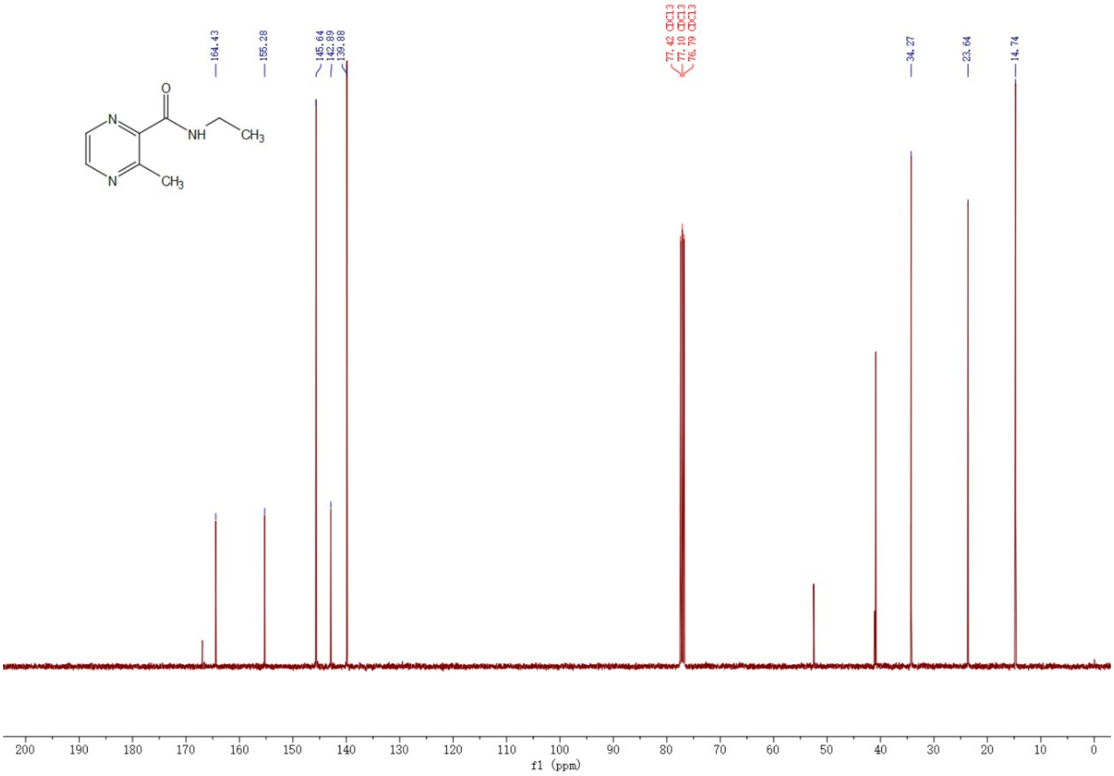

164.43
155.28
145.64
142.89
139.88
77.42 CDCl3
77.10 CDCl3
76.79 CDCl3
34.27
23.64
14.74
200 190 180 170 160 150 140 130 120 110 100 90 80 70 60 50 40 30 20 10 0
f1 (ppm)

3f

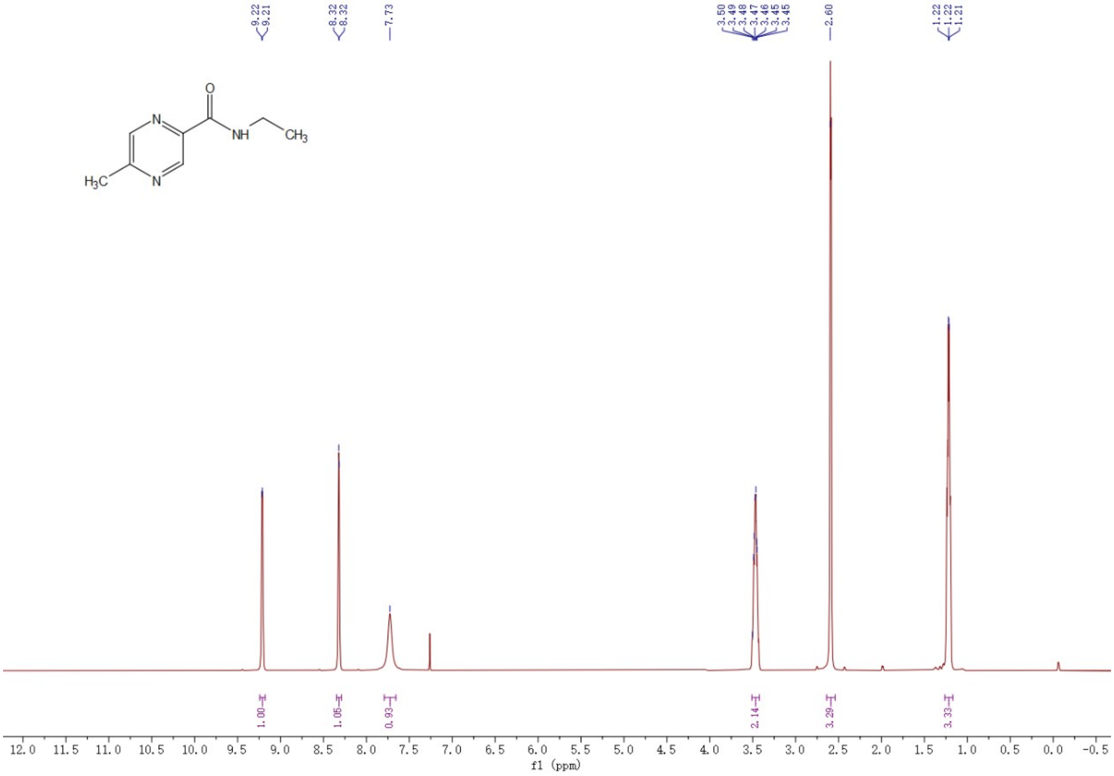

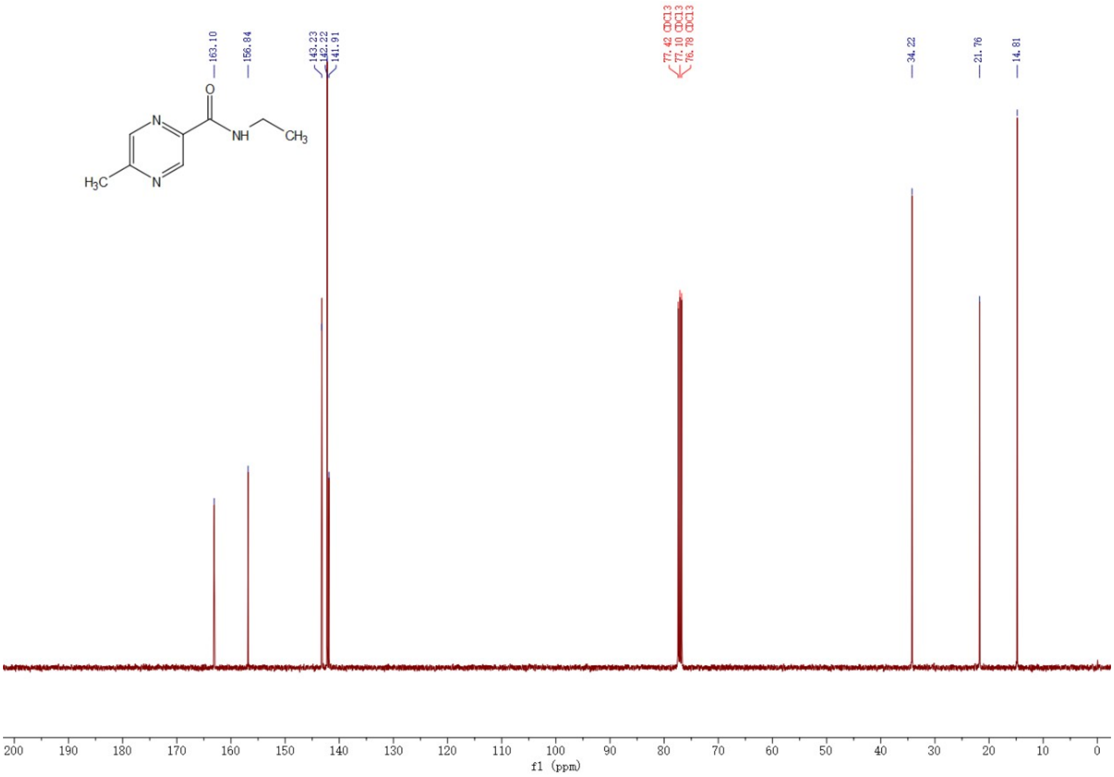

3g

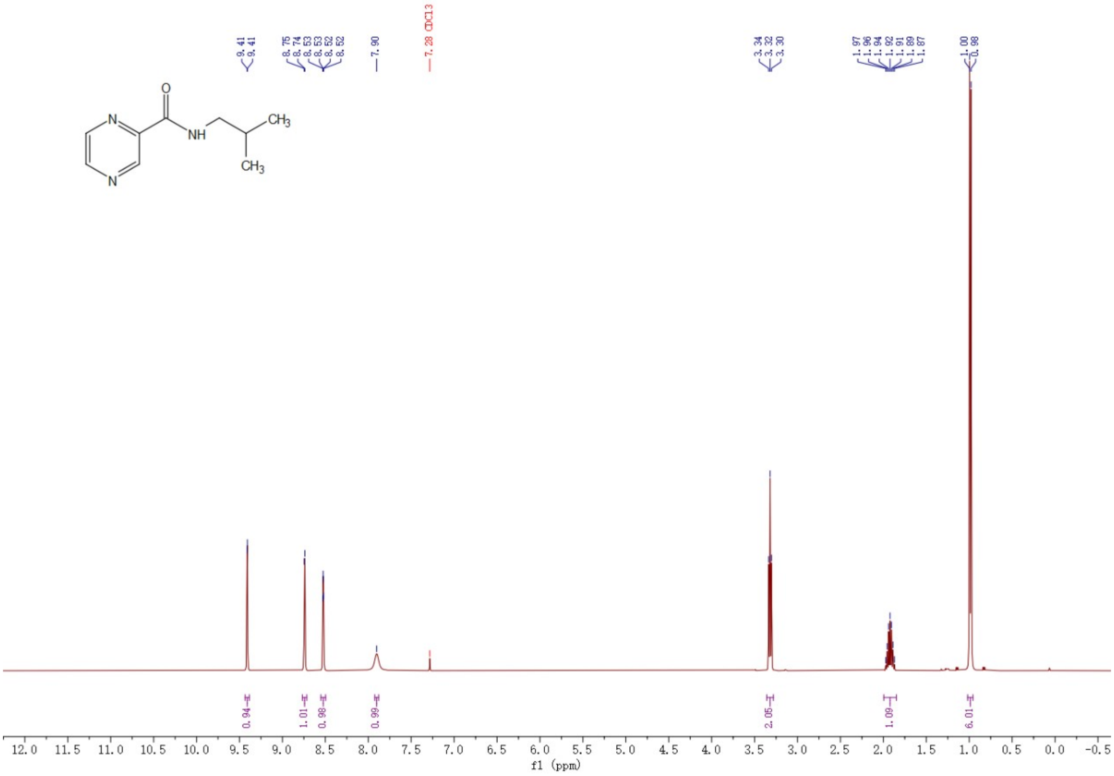
9.41
9.41
8.75
8.74
8.53
8.52
8.52
7.90
7.28 CDCl3
3.34
3.32
3.30
1.97
1.96
1.94
1.92
1.91
1.89
1.87
1.00
0.98
O
N
NH
CH3
CH3
N
0.94
1.01
0.98
0.99
2.05
1.09
6.01
12.0 11.5 11.0 10.5 10.0 9.5 9.0 8.5 8.0 7.5 7.0 6.5 6.0 5.5 5.0 4.5 4.0 3.5 3.0 2.5 2.0 1.5 1.0 0.5 0.0 -0.5
f1 (ppm)

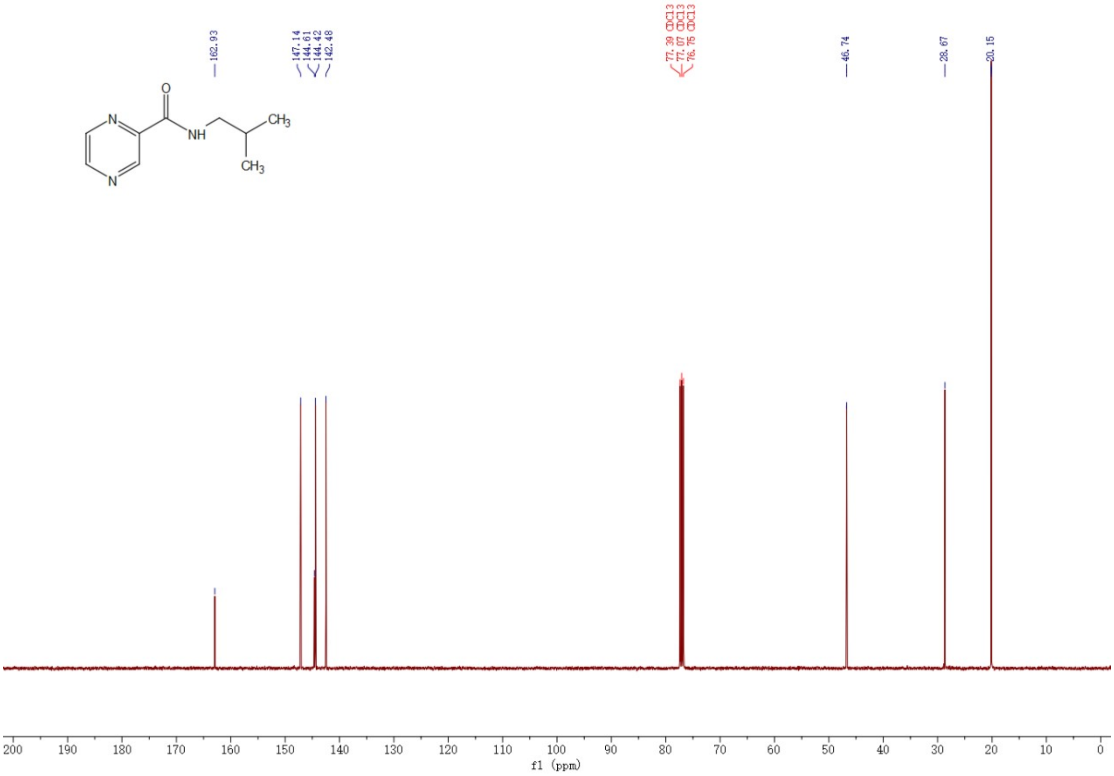
162.93
147.14
144.61
144.42
142.48
77.39 CDCl3
77.07 CDCl3
76.75 CDCl3
46.74
28.67
20.15
O
N
NH
CH3
CH3
N
200 190 180 170 160 150 140 130 120 110 100 90 80 70 60 50 40 30 20 10 0
f1 (ppm)

3h

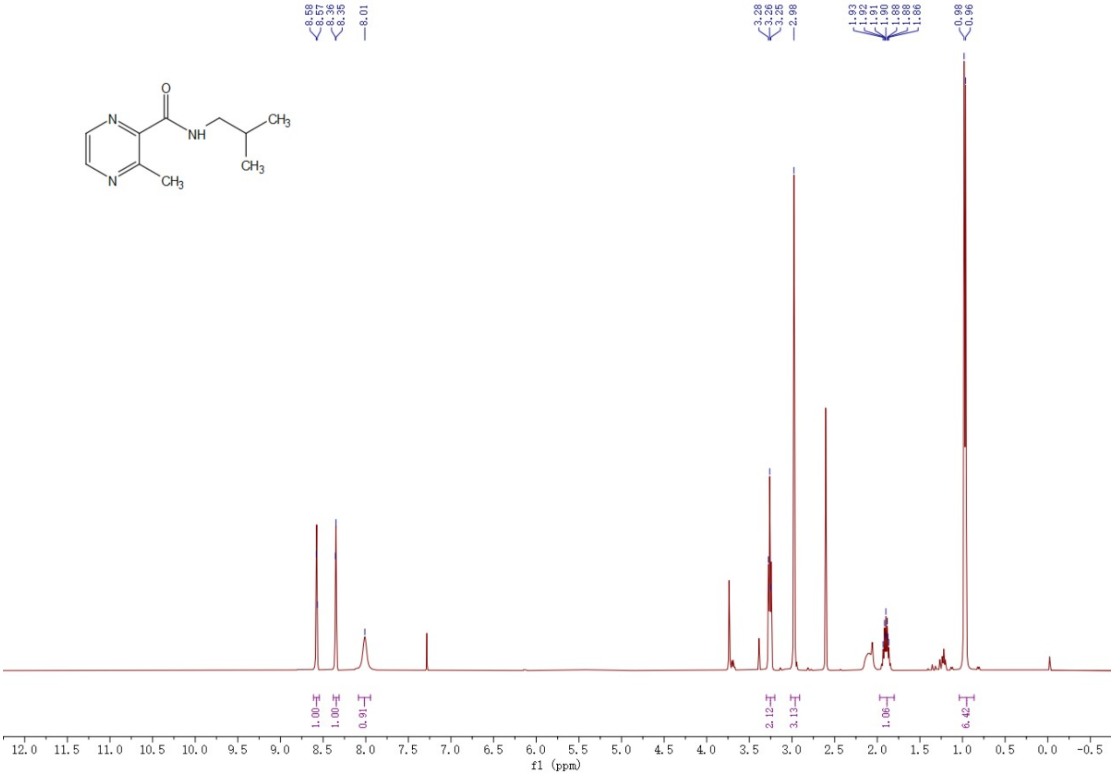

8.58
8.57
8.36
8.35
8.01
3.28
3.26
3.25
2.98
1.93
1.92
1.91
1.90
1.88
1.88
1.86
0.98
0.96
1.00
1.00
0.91
2.12
3.13
1.06
6.42
f1 (ppm)

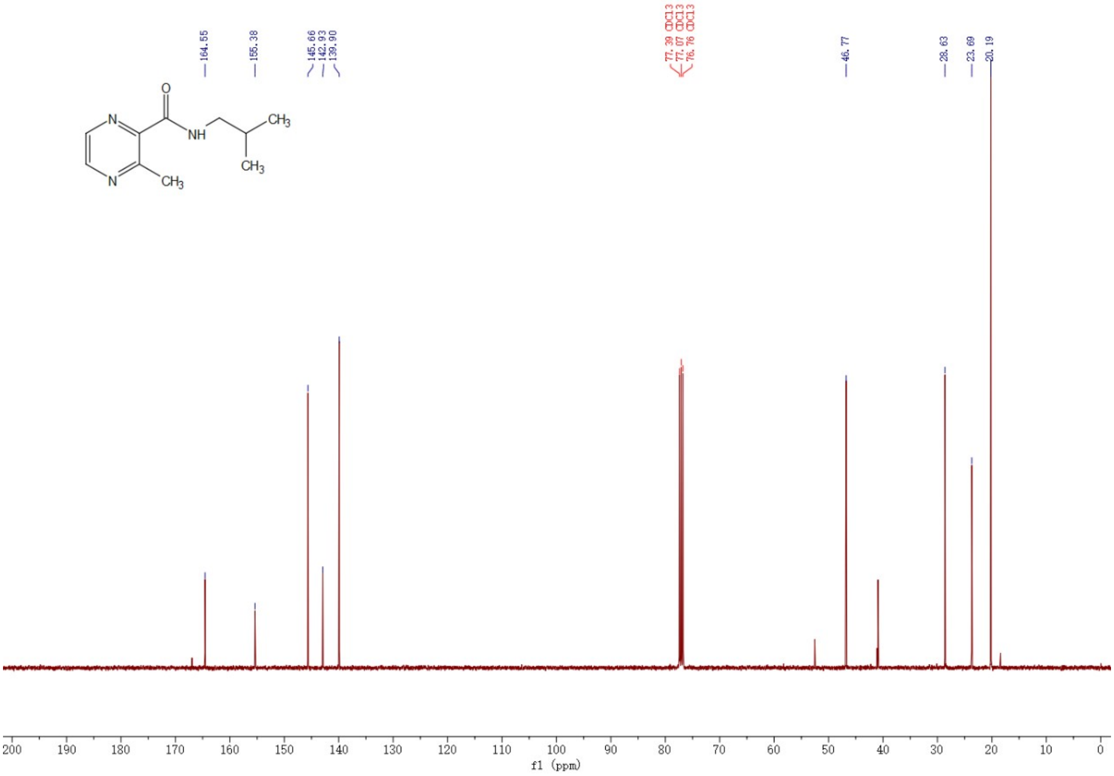

164.55
155.38
145.66
142.93
139.90
77.39 CDCl3
77.07 CDCl3
76.76 CDCl3
46.77
28.63
23.69
20.19
f1 (ppm)

3i

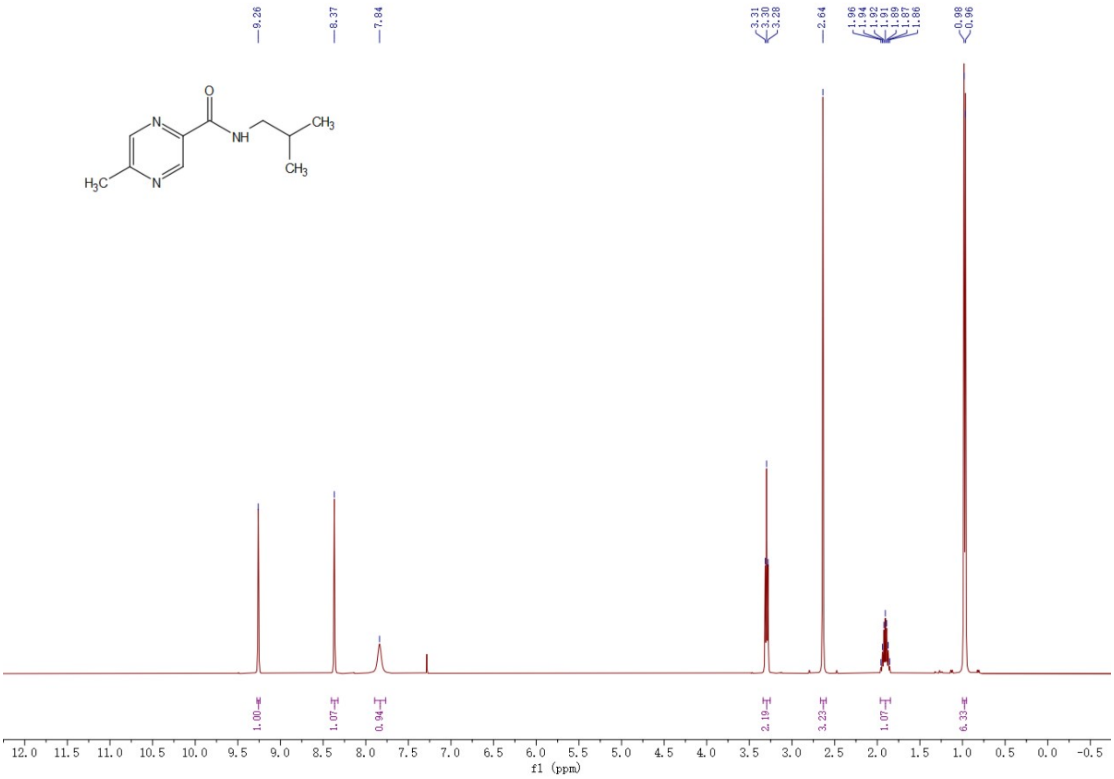
9.26
8.37
7.84
3.31
3.30
3.28
2.64
1.96
1.94
1.92
1.91
1.89
1.87
1.86
0.98
0.96
1.00
1.07
0.94
2.19
3.23
1.07
6.33
f1 (ppm)

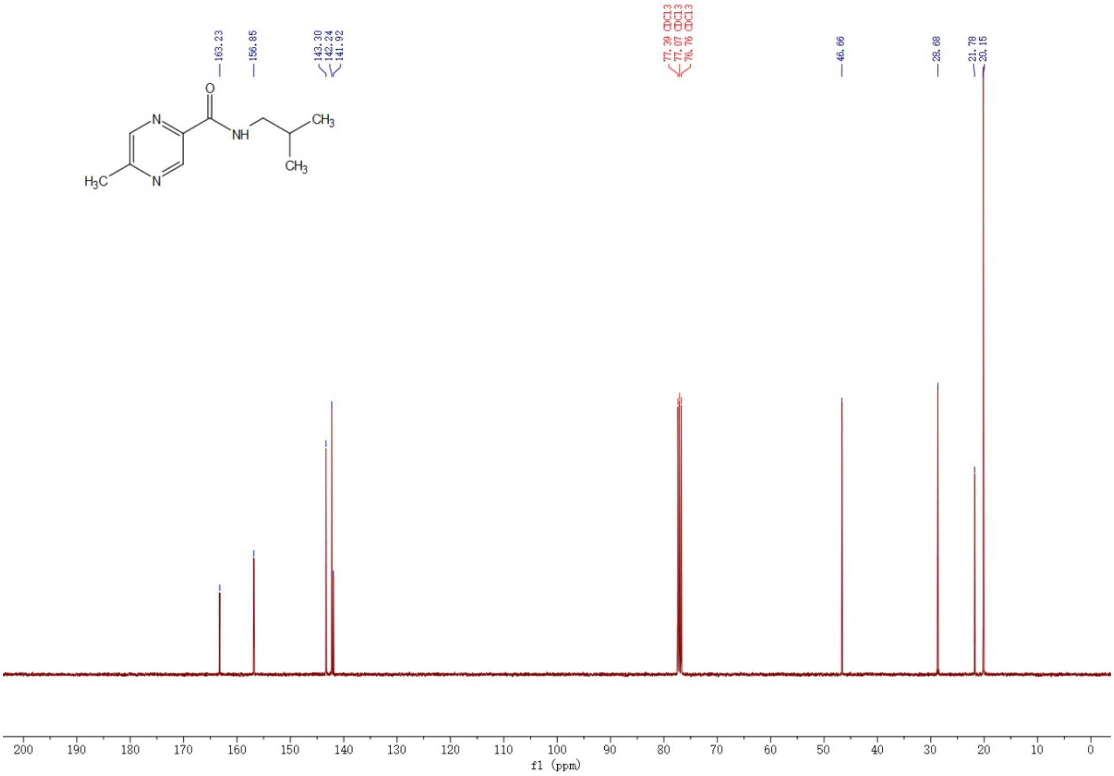
163.23
156.85
143.30
142.24
141.92
77.39 CDCl3
77.07 CDCl3
76.76 CDCl3
46.66
28.68
21.78
20.15
f1 (ppm)

3j

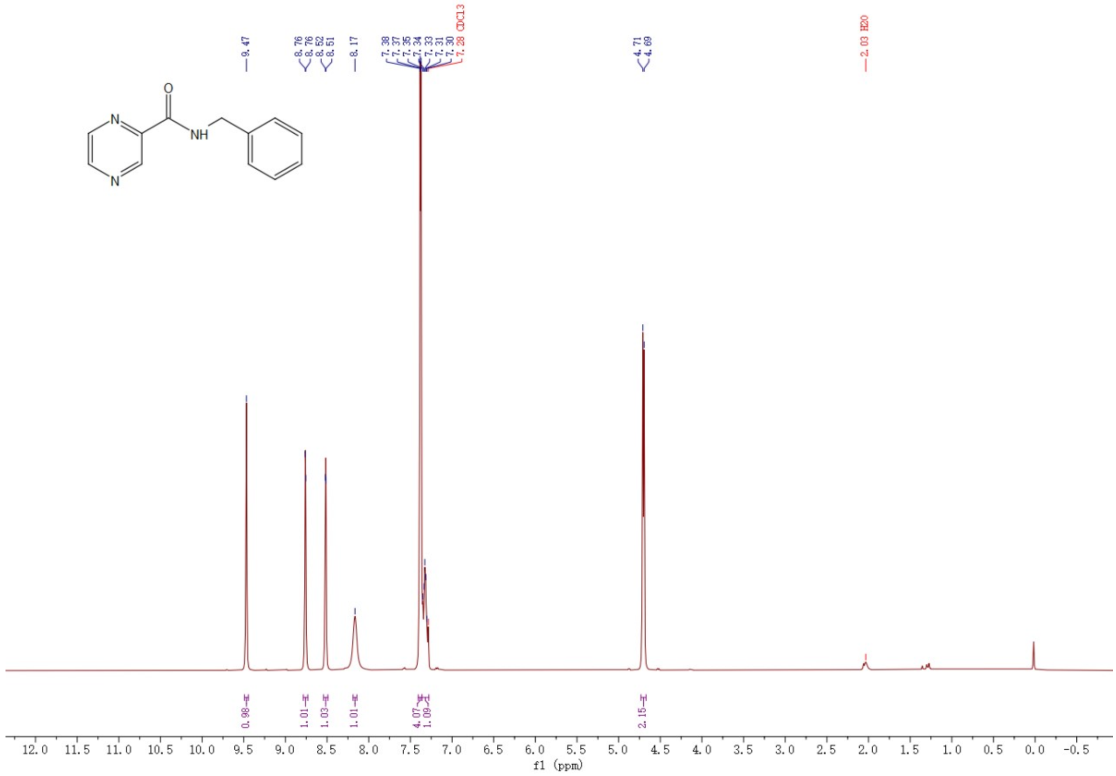

9.47
8.76
8.76
8.52
8.51
8.17
7.38
7.37
7.35
7.34
7.33
7.31
7.30
7.28 CDCl3
4.71
4.69
2.03 H2O
0.98
1.01
1.03
1.01
4.07
1.09
2.15
f1 (ppm)

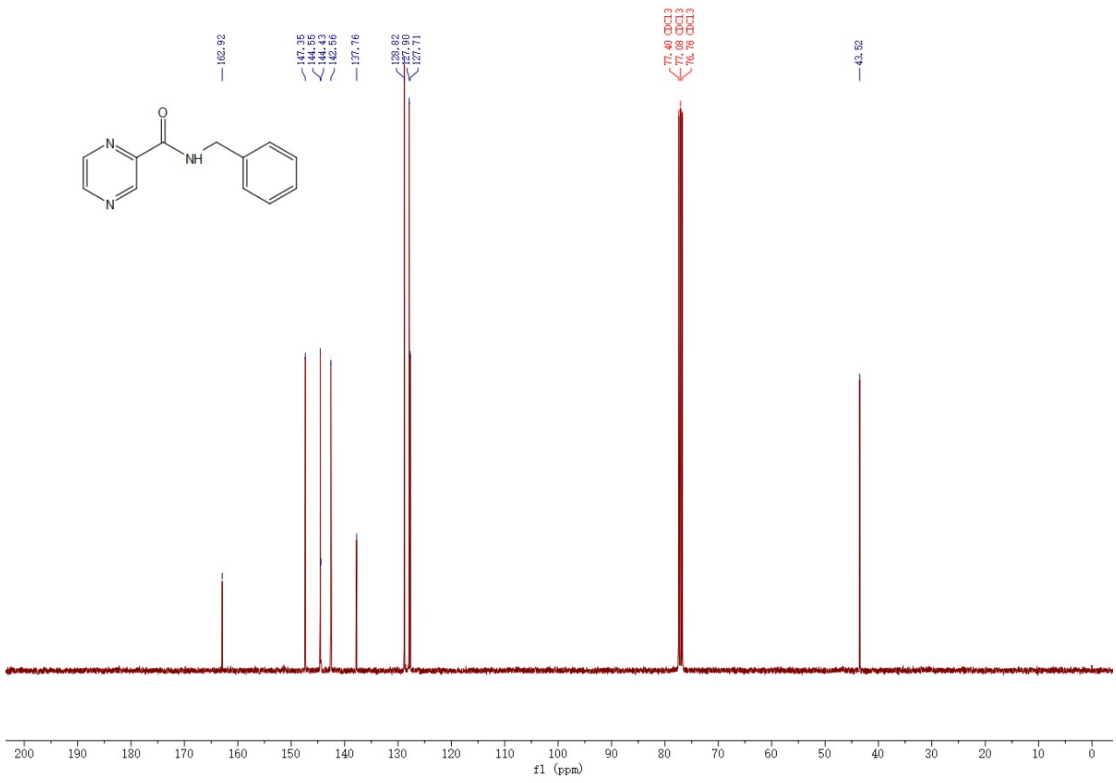

162.92
147.35
144.55
144.43
142.56
137.76
128.82
127.90
127.71
77.40 CDCl3
77.08 CDCl3
76.76 CDCl3
43.52
f1 (ppm)

3k

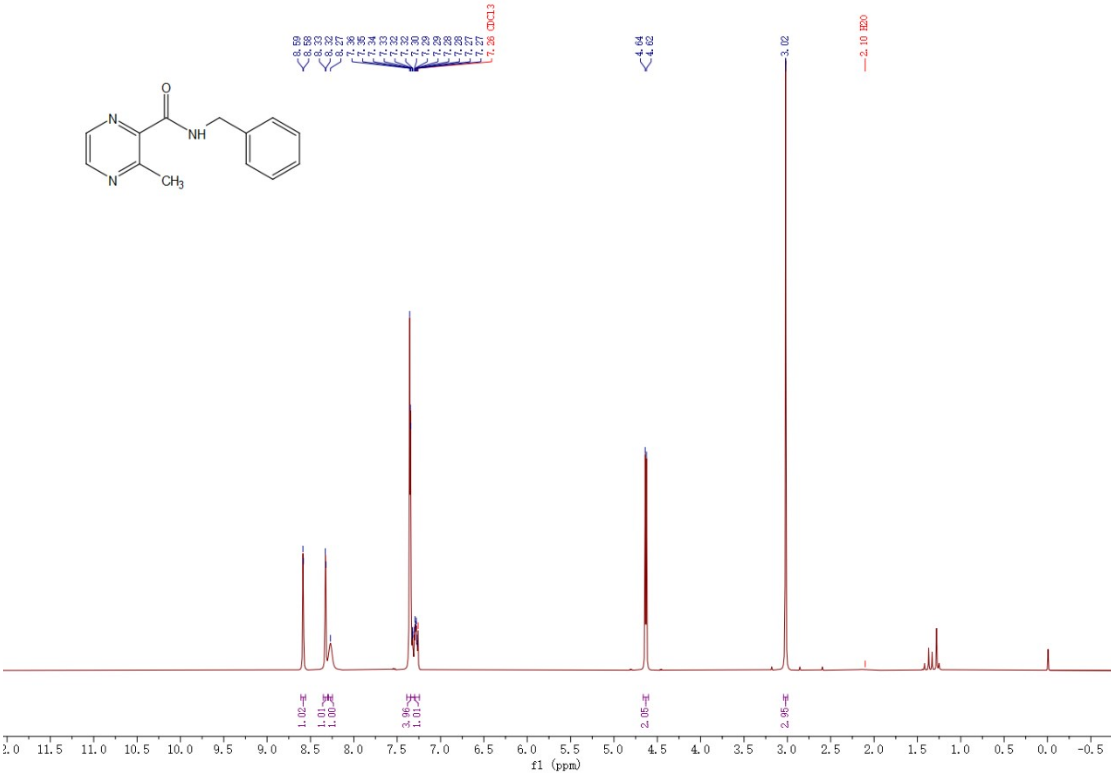
8.59
8.58
8.33
8.32
8.27
7.36
7.34
7.33
7.32
7.32
7.30
7.29
7.29
7.28
7.28
7.27
7.27
7.26 CDCl3
4.64
4.62
3.02
2.10 H2O
1.02
1.01
1.00
3.96
1.01
2.05
2.95
f1 (ppm)

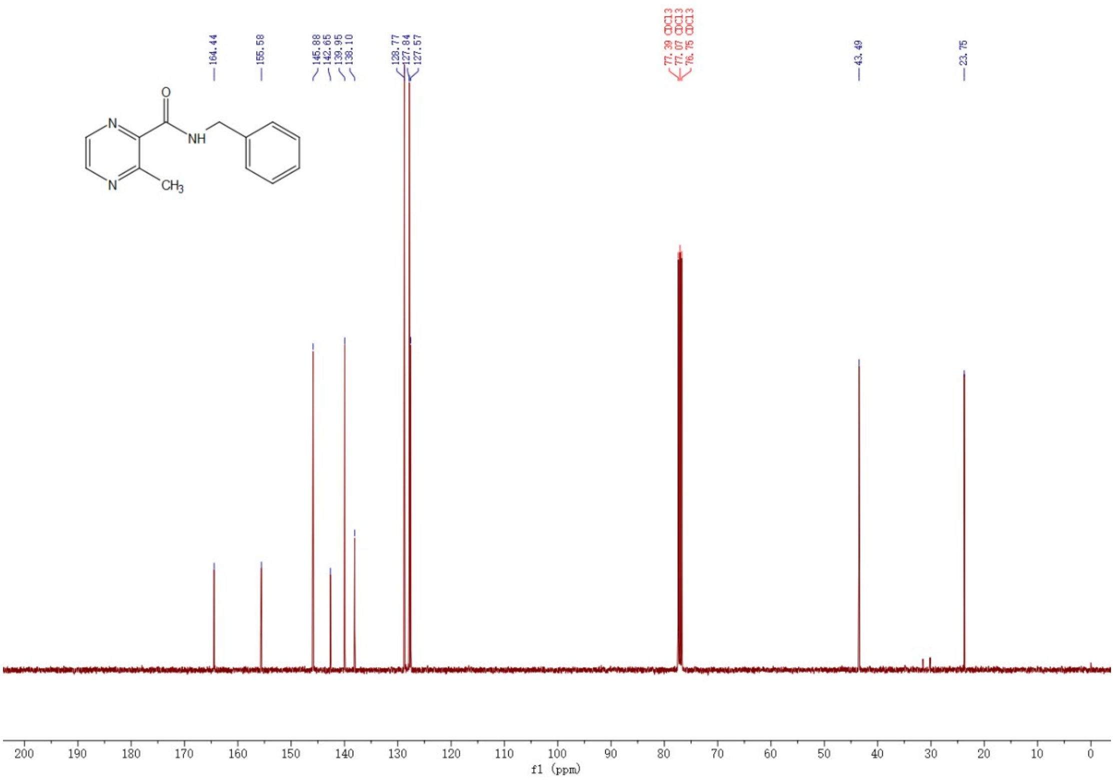
164.44
155.58
145.88
142.65
139.95
138.10
128.77
127.84
127.57
77.39 CDCl3
77.07 CDCl3
76.75 CDCl3
43.49
23.75
f1 (ppm)

31

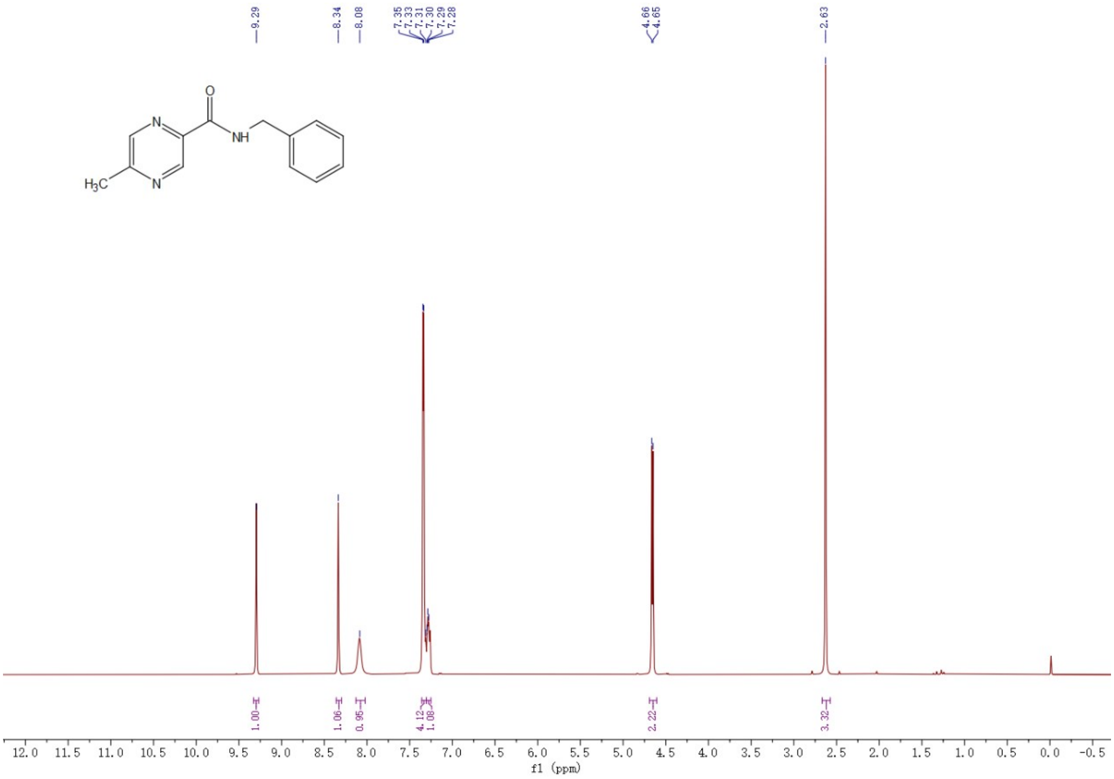

9.29
8.34
8.08
7.35
7.33
7.31
7.30
7.29
7.28
4.66
4.65
2.63
H3C
N
N
O
NH
1.00
1.06
0.95
4.12
1.08
2.22
3.32
12.0 11.5 11.0 10.5 10.0 9.5 9.0 8.5 8.0 7.5 7.0 6.5 6.0 5.5 5.0 4.5 4.0 3.5 3.0 2.5 2.0 1.5 1.0 0.5 0.0 -0.5
f1 (ppm)

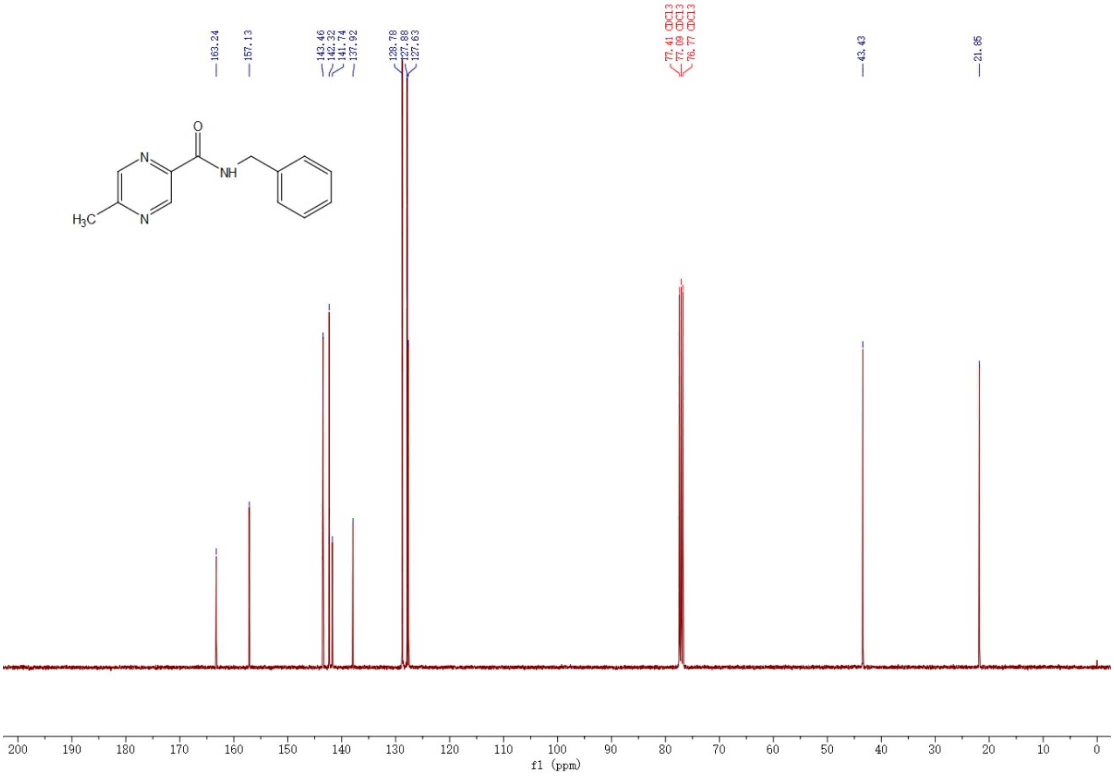

163.24
157.13
143.46
142.32
141.74
137.92
128.78
127.88
127.63
77.41 CDCl3
77.09 CDCl3
76.77 CDCl3
43.43
21.85
H3C
N
N
O
NH
200 190 180 170 160 150 140 130 120 110 100 90 80 70 60 50 40 30 20 10 0
f1 (ppm)

3m

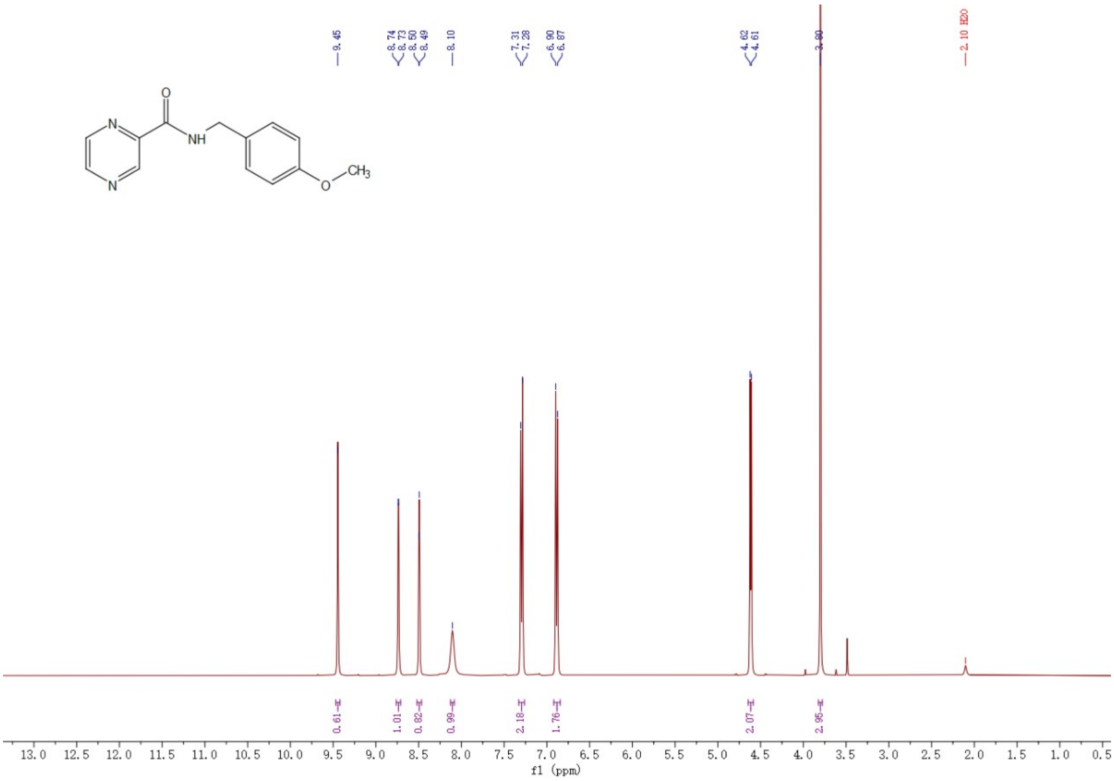

9.45
8.74
8.73
8.50
8.49
8.10
7.31
7.28
6.90
6.87
4.62
4.61
3.80
2.10 H2O
O
N
NH
N
O
CH3
0.61
1.01
0.82
0.99
2.18
1.76
2.07
2.95
13.0 12.5 12.0 11.5 11.0 10.5 10.0 9.5 9.0 8.5 8.0 7.5 7.0 6.5 6.0 5.5 5.0 4.5 4.0 3.5 3.0 2.5 2.0 1.5 1.0 0.5
f1 (ppm)

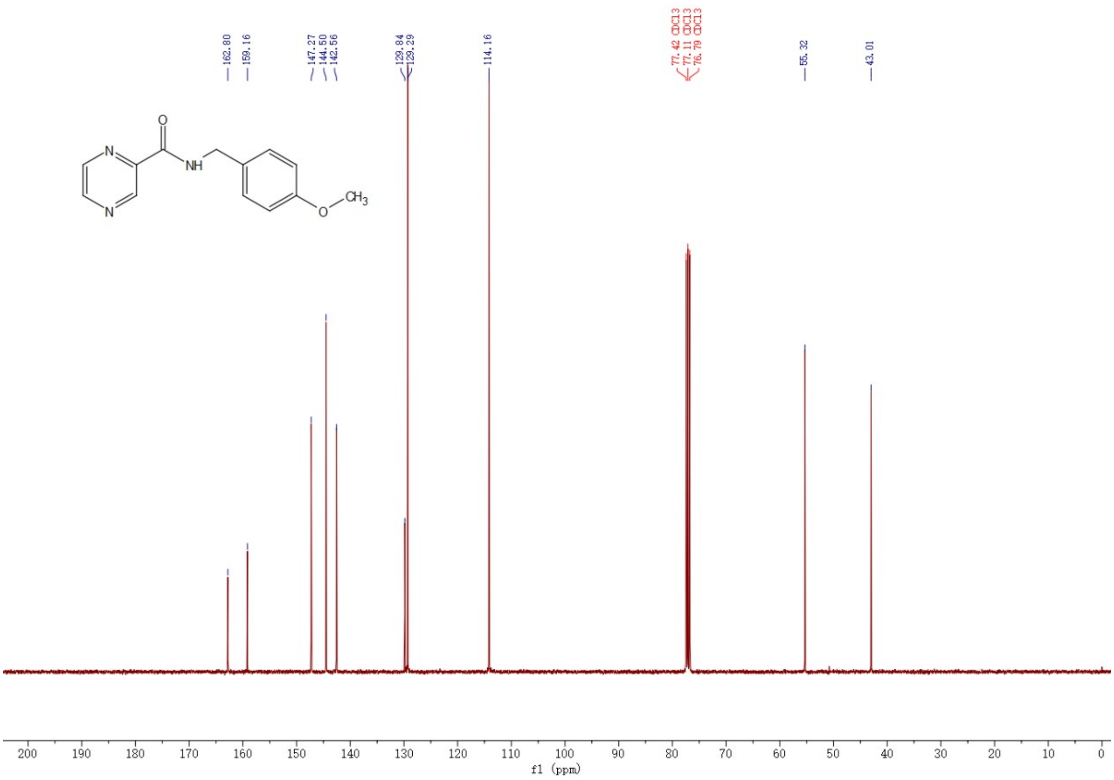

162.80
159.16
147.27
144.50
142.56
129.84
129.29
114.16
77.42 CDCl3
77.11 CDCl3
76.79 CDCl3
55.32
43.01
O
N
NH
N
O
CH3
200 190 180 170 160 150 140 130 120 110 100 90 80 70 60 50 40 30 20 10 0
f1 (ppm)

3n

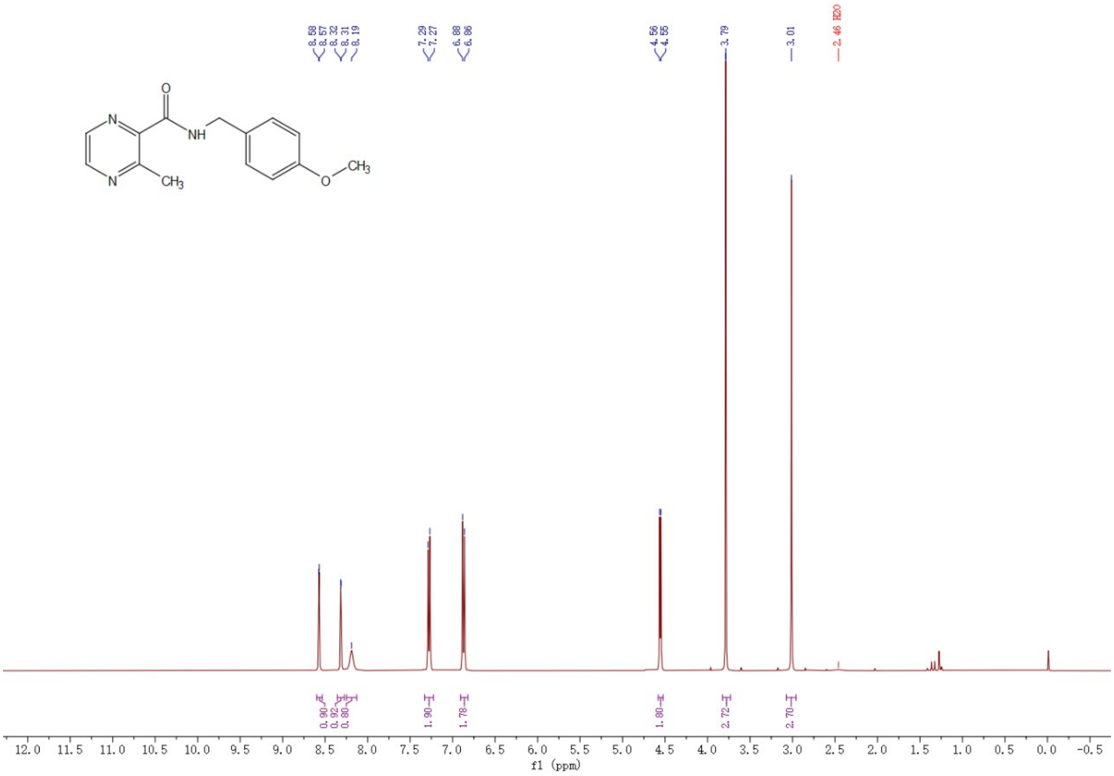

8.58
8.57
8.32
8.31
8.19
7.29
7.27
6.88
6.86
4.56
4.55
3.79
3.01
2.46 H2O
0.90
0.92
0.80
1.90
1.78
1.80
2.72
2.70
f1 (ppm)

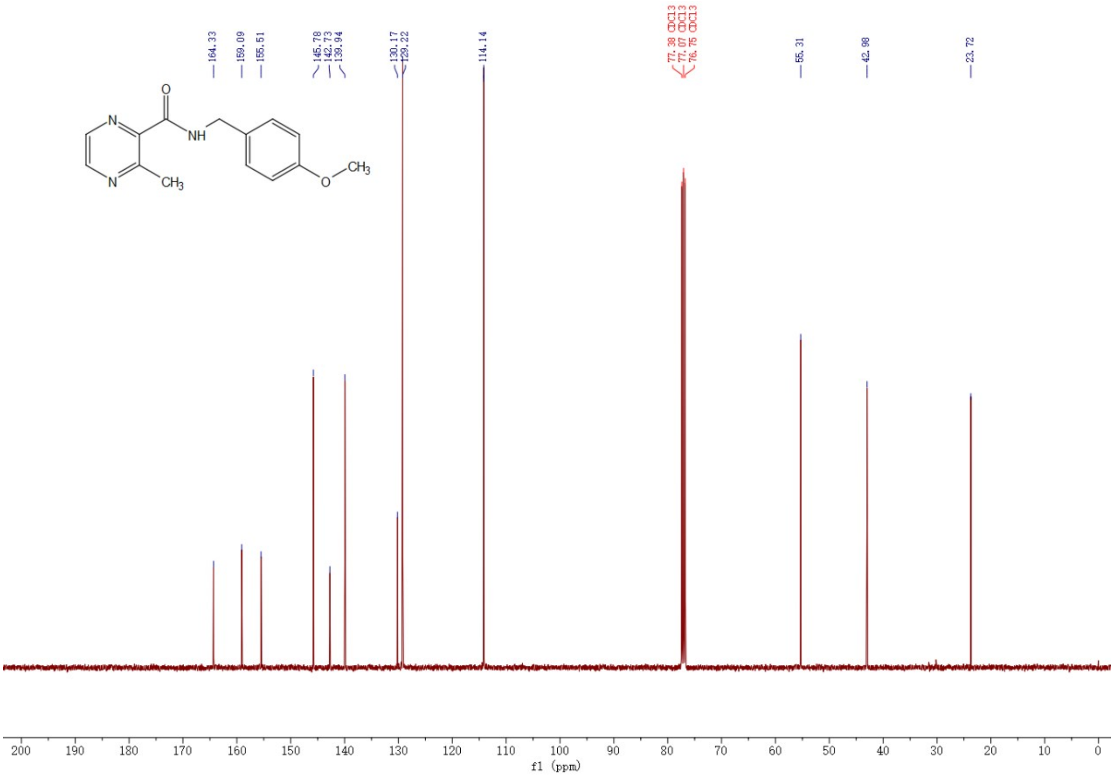

164.33
159.09
155.51
145.78
142.73
139.94
130.17
129.22
114.14
77.38 CDCl3
77.07 CDCl3
76.75 CDCl3
55.31
42.98
23.72
f1 (ppm)

3o

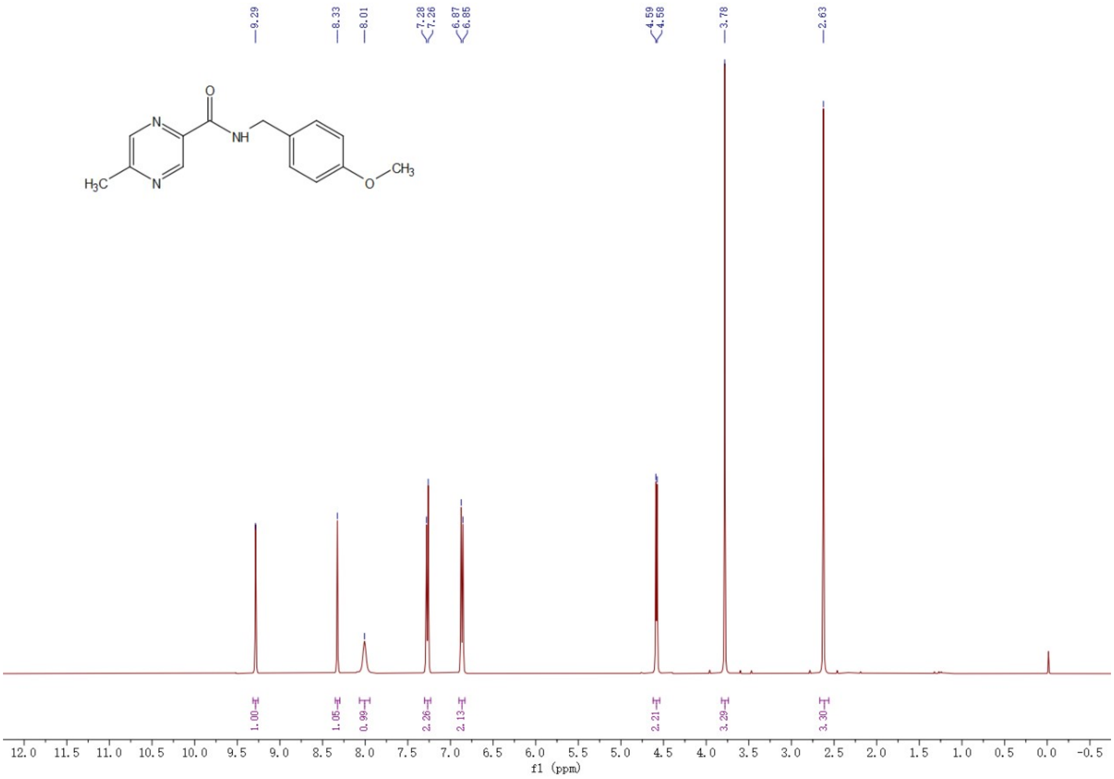

9.29
8.33
8.01
7.28
7.26
6.87
6.85
4.59
4.58
3.78
2.63
H3C
N
N
O
NH
O
CH3
1.00
1.05
0.99
2.26
2.13
2.21
3.29
3.30
12.0 11.5 11.0 10.5 10.0 9.5 9.0 8.5 8.0 7.5 7.0 6.5 6.0 5.5 5.0 4.5 4.0 3.5 3.0 2.5 2.0 1.5 1.0 0.5 0.0 -0.5
f1 (ppm)

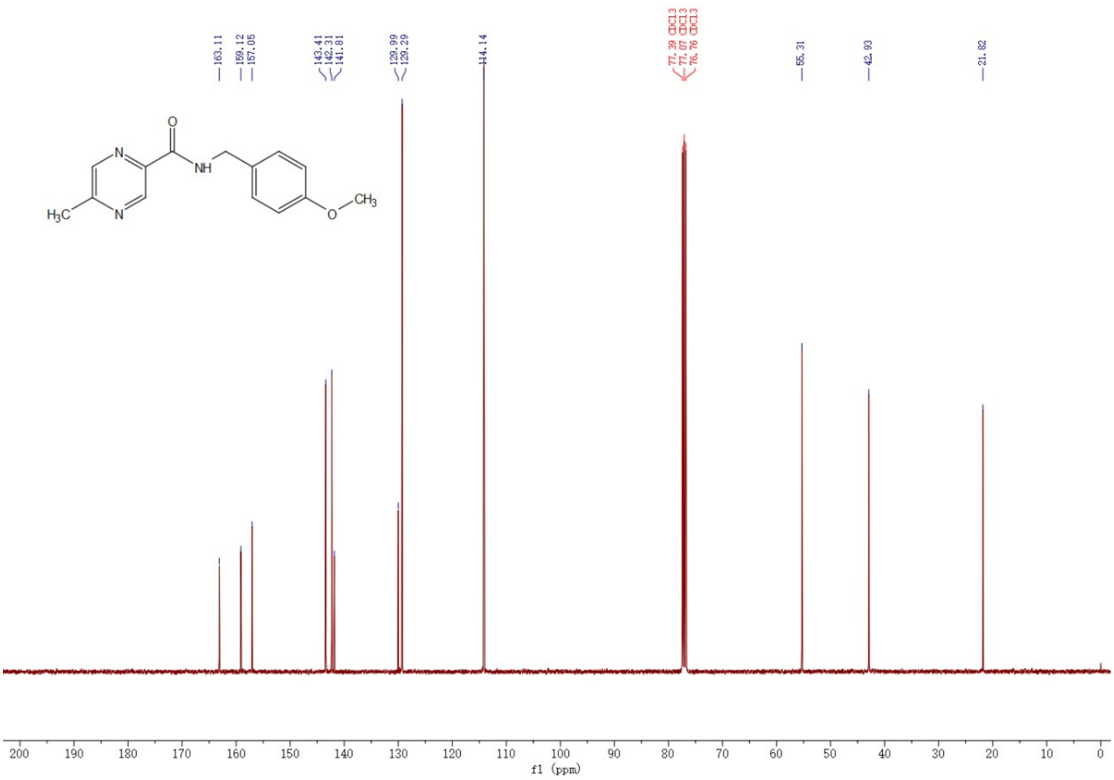

163.11
159.12
157.05
143.41
142.31
141.81
129.99
129.29
114.14
77.39 CDCl3
77.07 CDCl3
76.76 CDCl3
55.31
42.93
21.82
H3C
N
N
O
NH
O
CH3
200 190 180 170 160 150 140 130 120 110 100 90 80 70 60 50 40 30 20 10 0
f1 (ppm)

3p

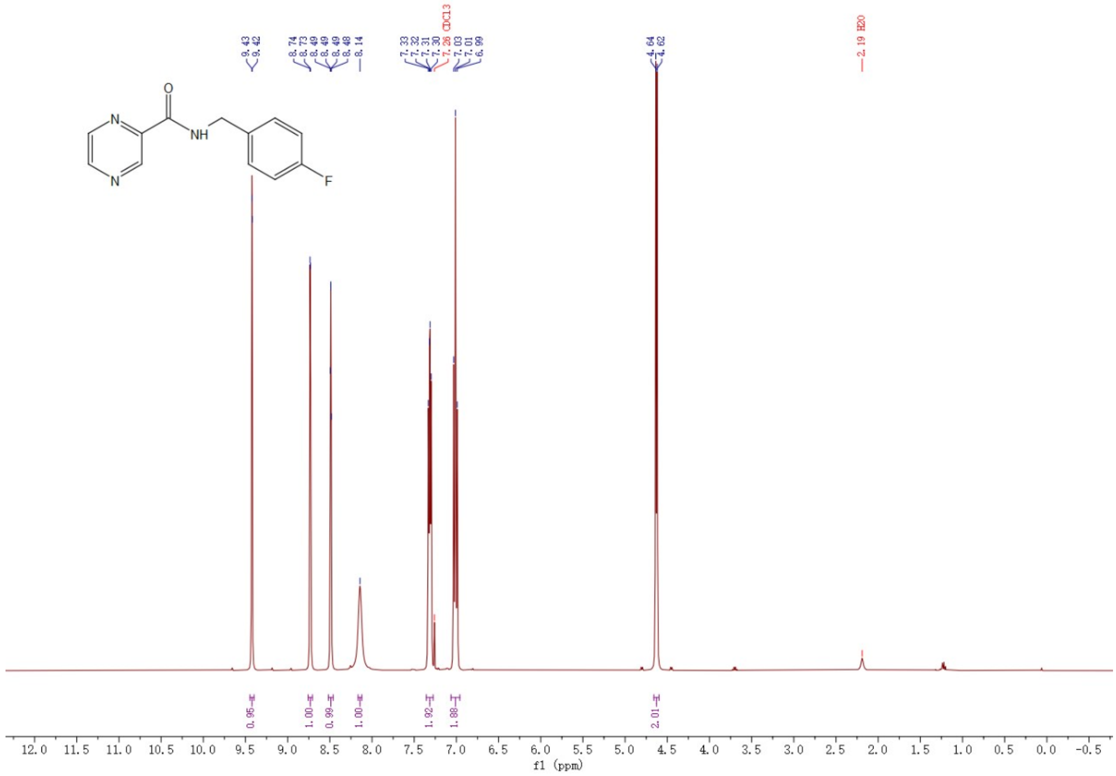

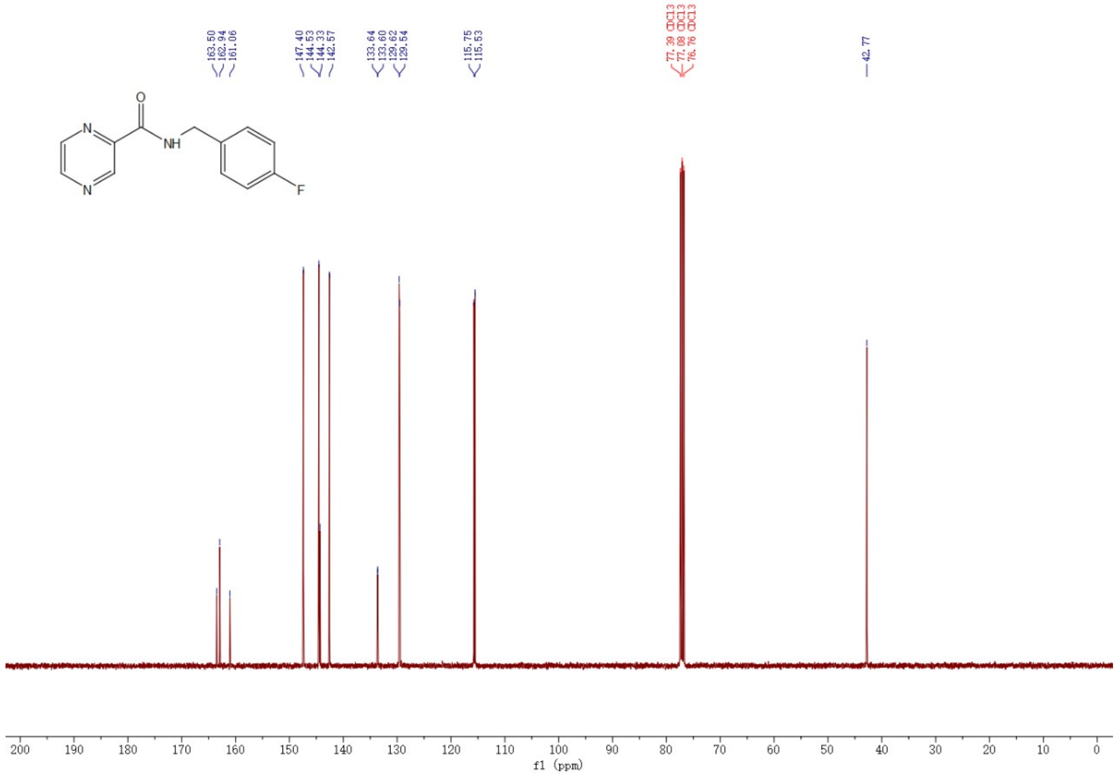

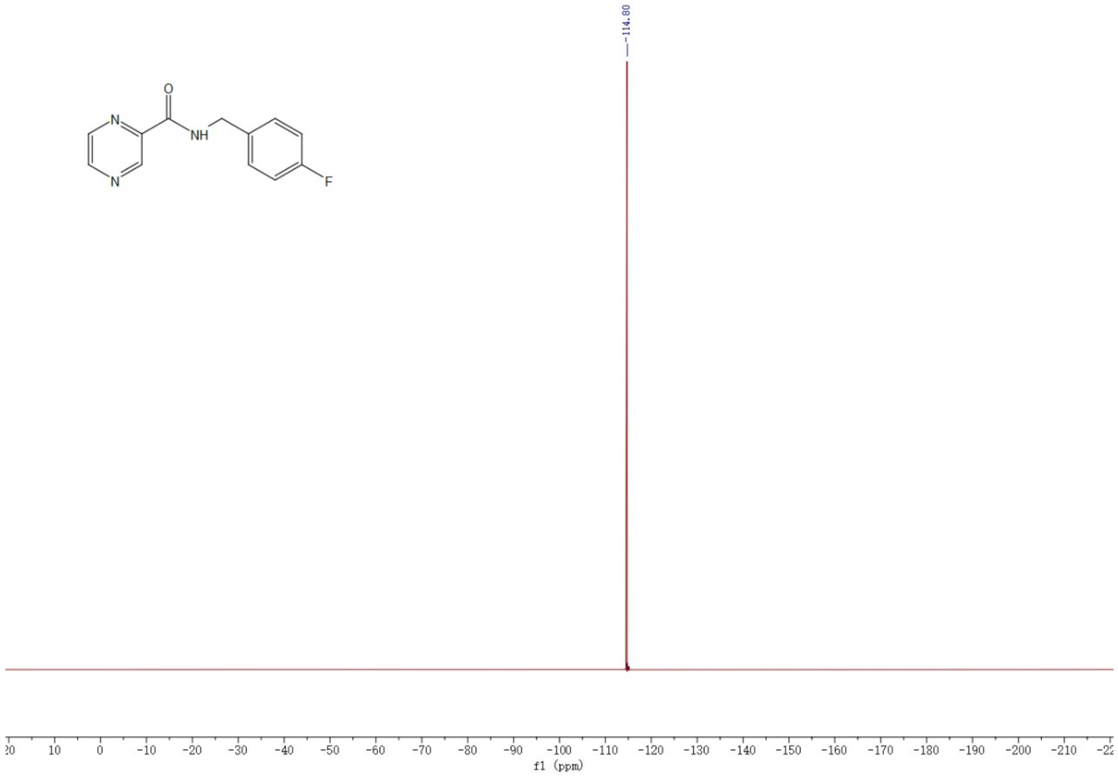

-114.80
O
N
NH
N
F
20 10 0 -10 -20 -30 -40 -50 -60 -70 -80 -90 -100 -110 -120 -130 -140 -150 -160 -170 -180 -190 -200 -210 -22
f1 (ppm)

3q

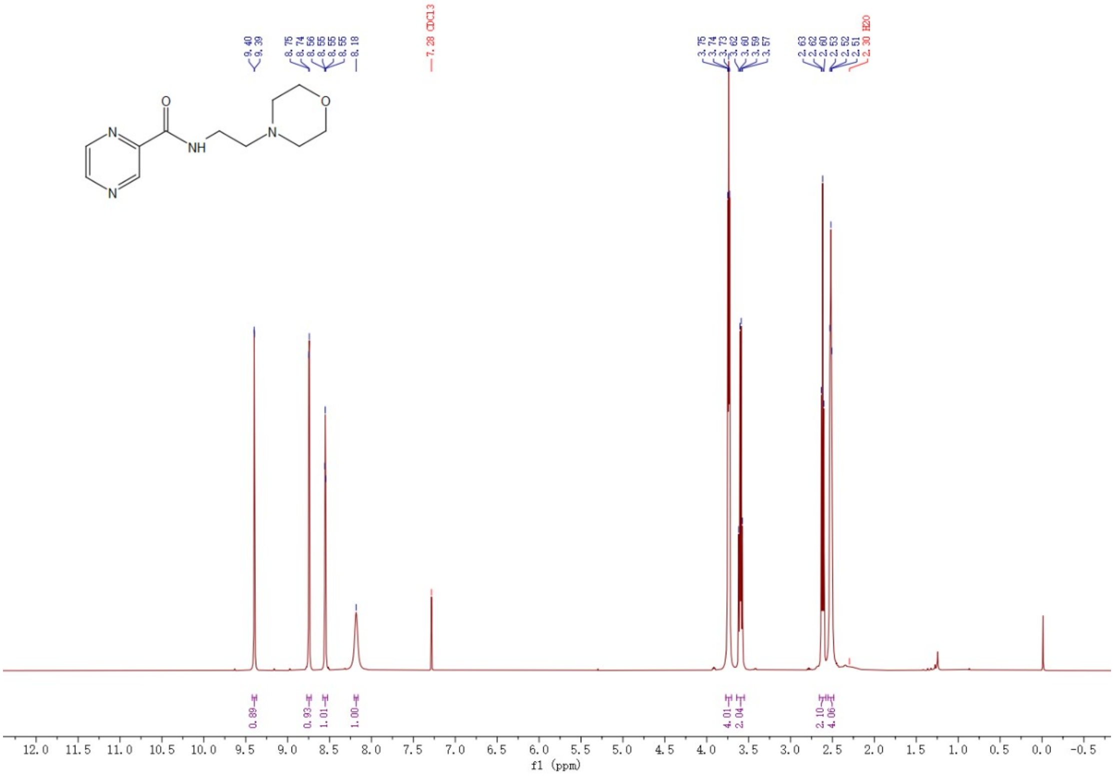

9.40
9.39
8.75
8.74
8.56
8.55
8.55
8.55
8.18
7.28 CDCl3
3.75
3.74
3.73
3.62
3.60
3.59
3.57
2.63
2.62
2.60
2.53
2.52
2.51
2.30 H2O
0.89
0.93
1.01
1.00
4.01
2.04
2.10
4.06
f1 (ppm)

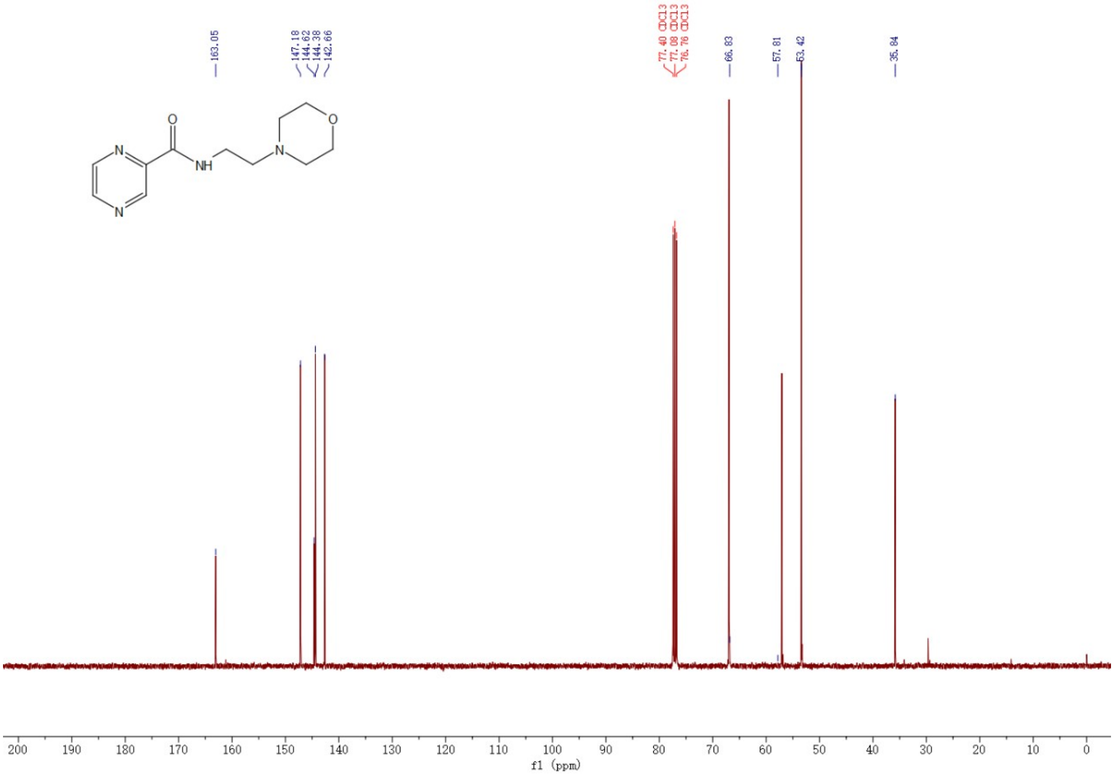

163.05
147.18
144.62
144.38
142.66
77.40 CDCl3
77.08 CDCl3
76.76 CDCl3
66.83
57.81
53.42
35.84
f1 (ppm)

3r

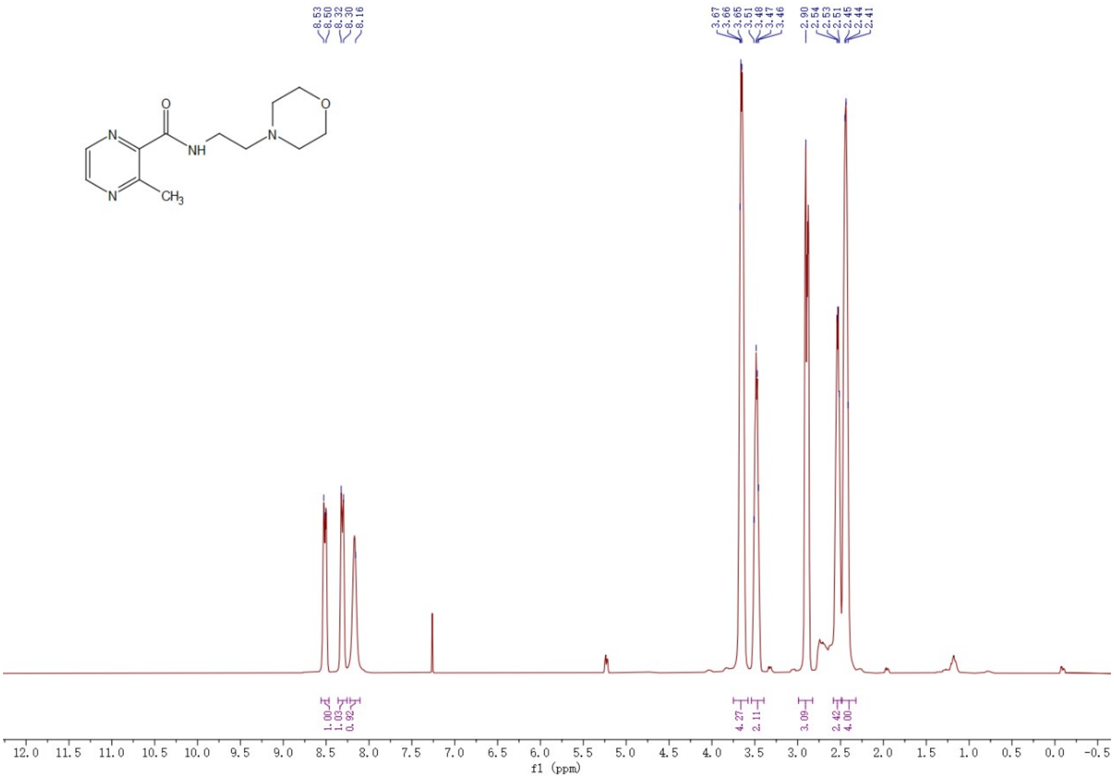
8.53
8.50
8.32
8.30
8.16
3.67
3.66
3.65
3.51
3.48
3.47
3.46
2.90
2.54
2.53
2.51
2.45
2.44
2.41
1.00
1.03
0.92
4.27
2.11
3.09
2.42
4.00
f1 (ppm)

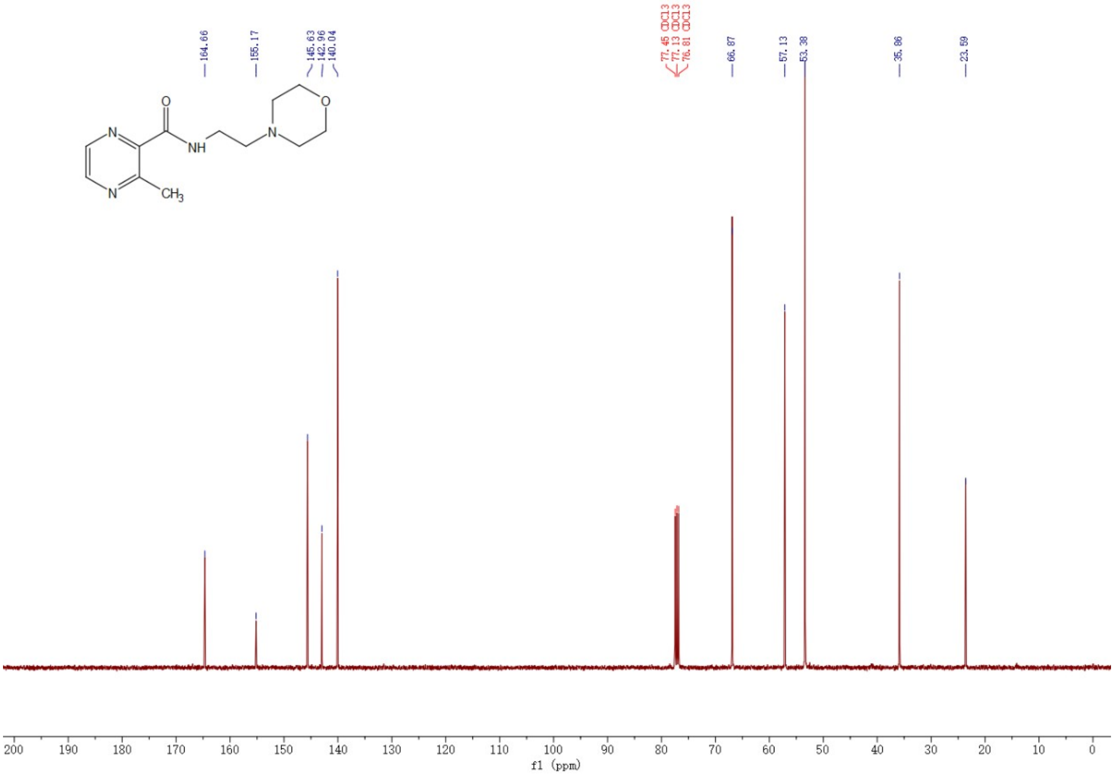
164.66
155.17
145.63
142.96
140.04
77.45 CDCl3
77.13 CDCl3
76.81 CDCl3
66.87
57.13
53.38
35.86
23.59
f1 (ppm)

3s

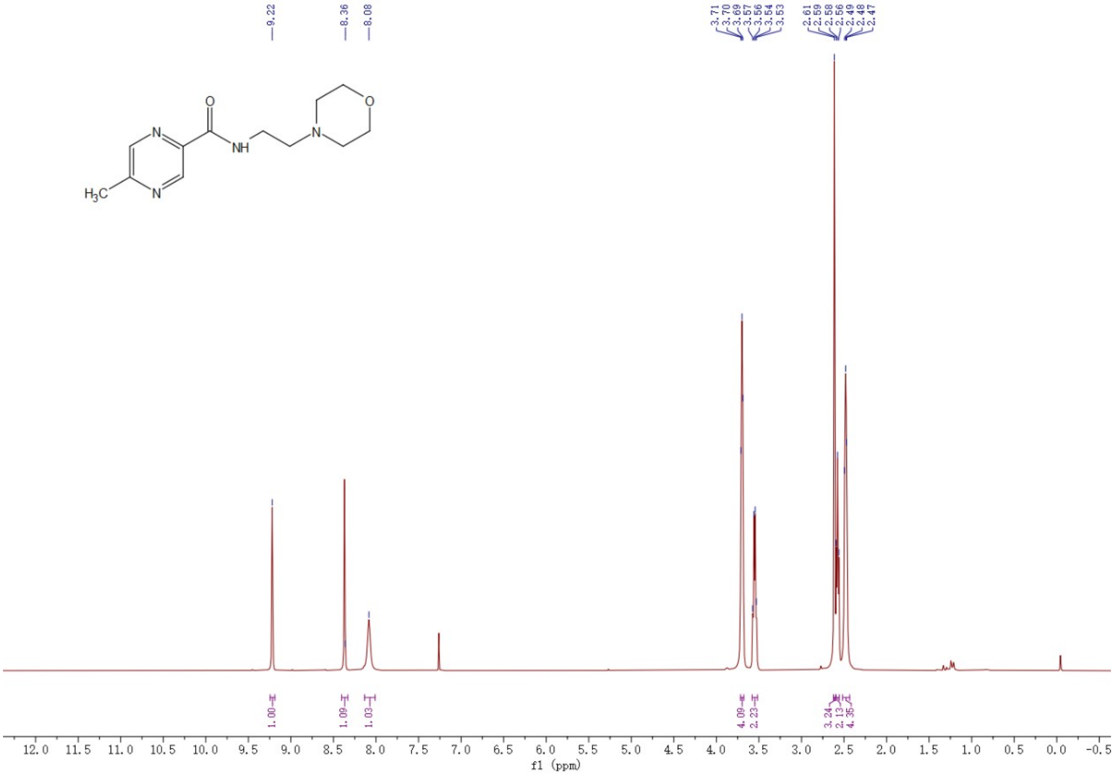

9.22
8.36
8.08
3.71
3.70
3.69
3.57
3.56
3.54
3.53
2.61
2.59
2.58
2.56
2.49
2.48
2.47
O
N
NH
N
N
O
H3C
1.00
1.09
1.03
4.09
2.23
3.24
2.13
4.35
f1 (ppm)

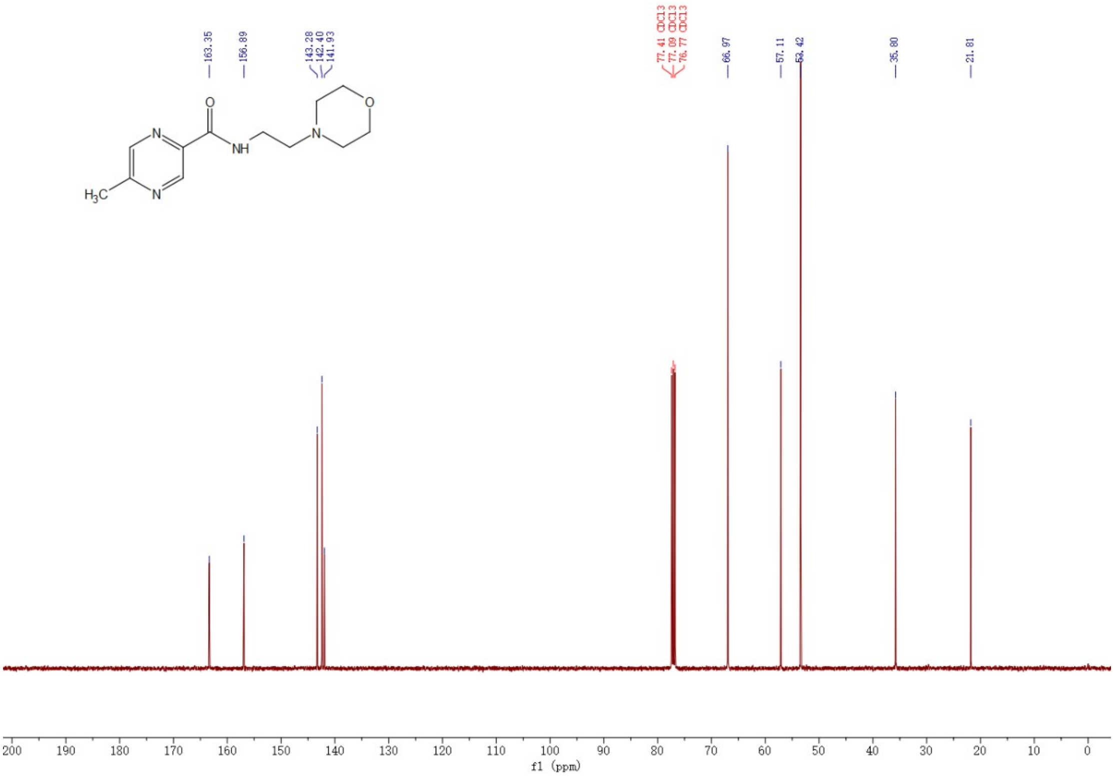

163.35
156.89
143.28
142.40
141.93
77.41 CDCl3
77.09 CDCl3
76.77 CDCl3
66.97
57.11
53.42
35.80
21.81
O
N
NH
N
N
O
H3C
f1 (ppm)

3t

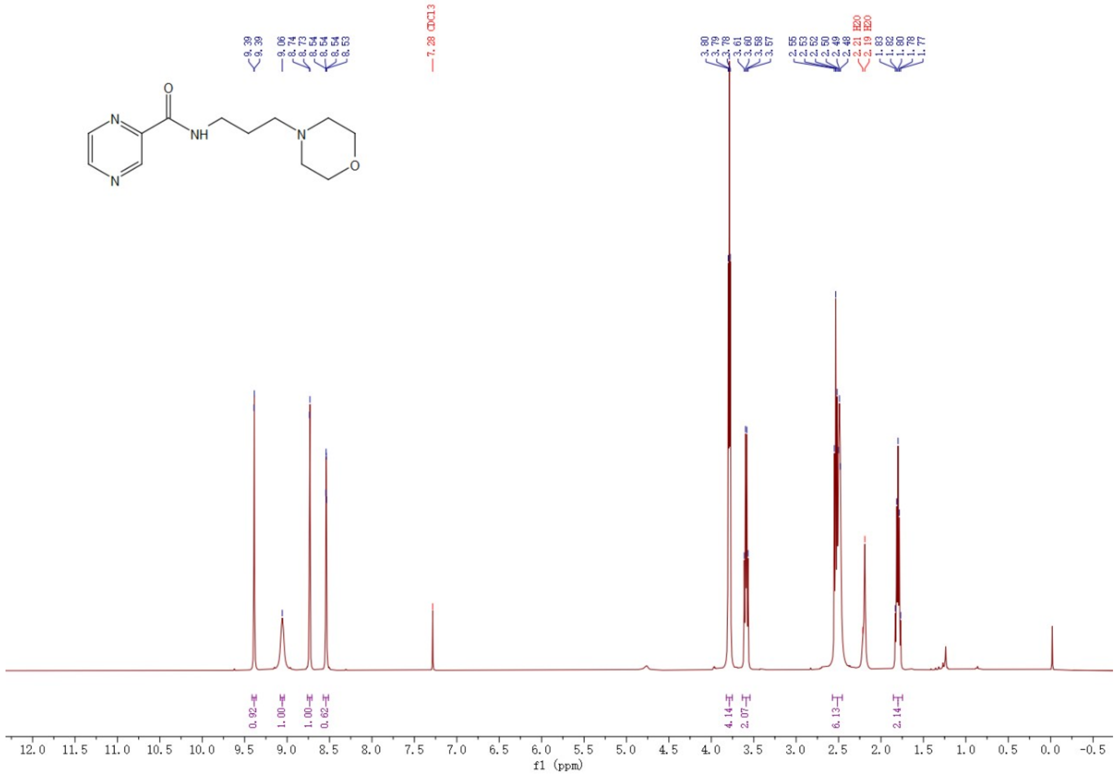

9.39
9.39
9.06
8.74
8.73
8.54
8.54
8.54
8.53
7.28 CDCl3
3.80
3.79
3.78
3.61
3.60
3.58
3.57
2.55
2.53
2.52
2.50
2.49
2.48
2.21 H2O
2.19 H2O
1.83
1.82
1.80
1.78
1.77
N
N
O
NH
N
O
0.92
1.00
1.00
0.62
4.14
2.07
6.13
2.14
f1 (ppm)

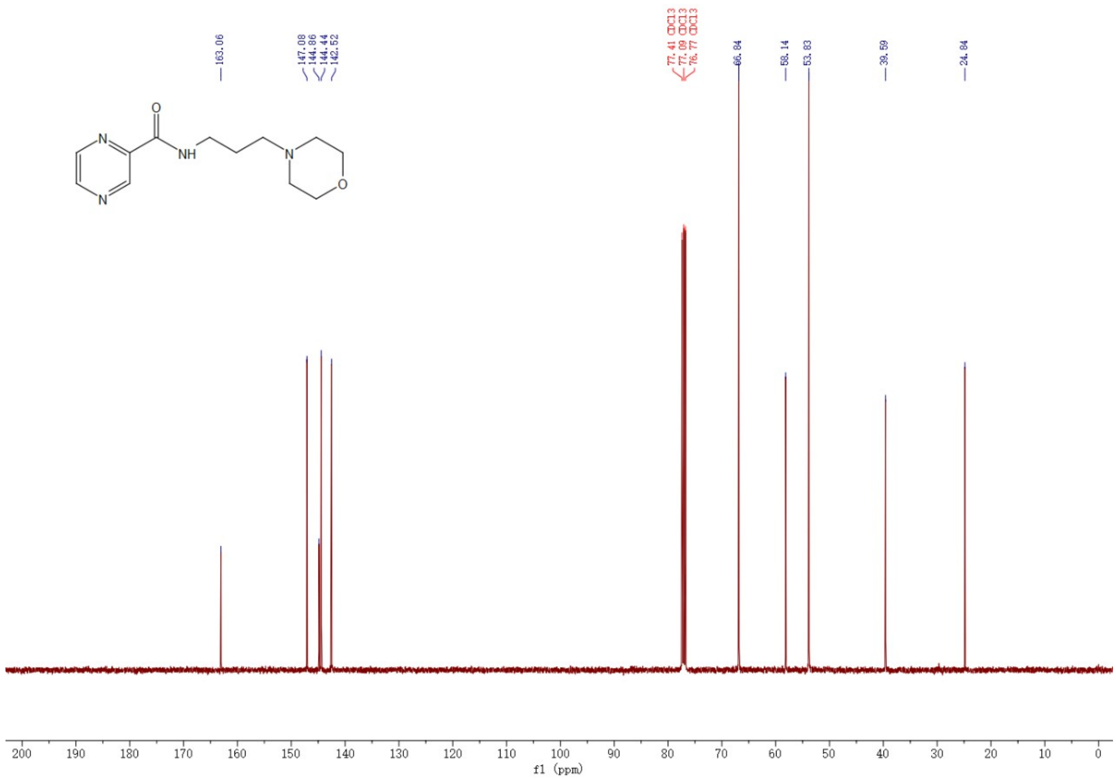

163.06
147.08
144.86
144.44
142.52
77.41 CDCl3
77.09 CDCl3
76.77 CDCl3
66.84
58.14
53.83
39.59
24.84
N
N
O
NH
N
O
f1 (ppm)

3u

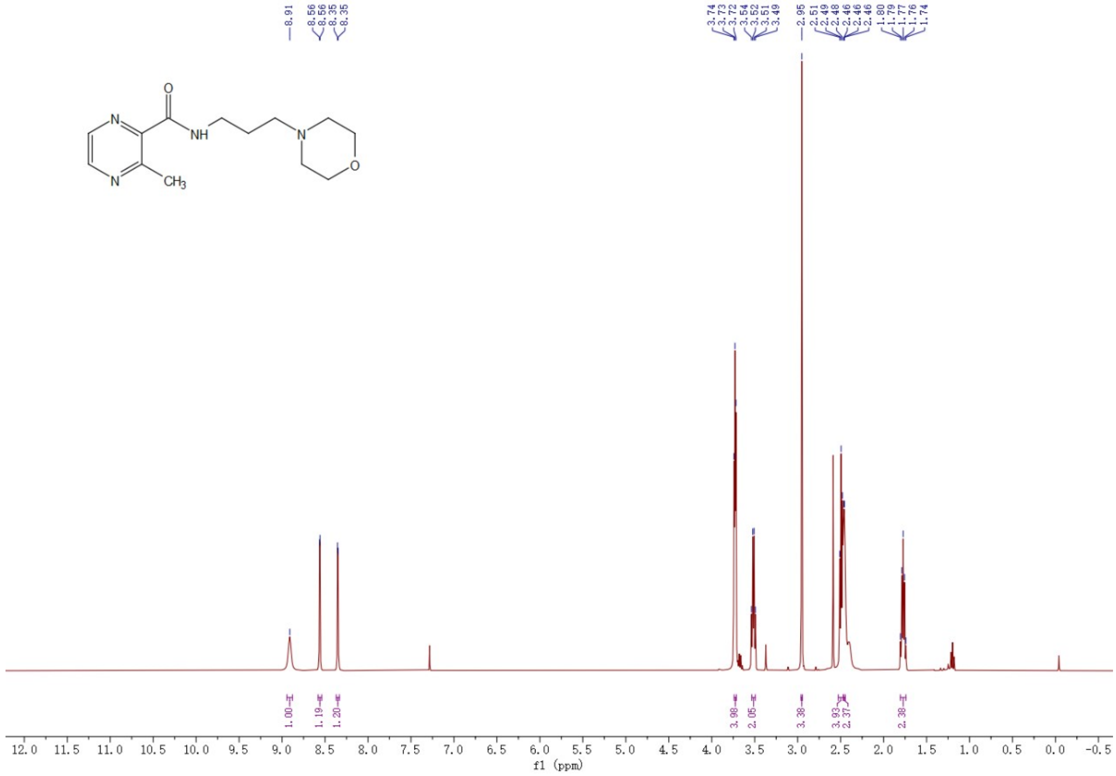
8.91
8.56
8.56
8.35
8.35
3.74
3.73
3.72
3.54
3.52
3.51
3.49
2.95
2.51
2.49
2.48
2.46
2.46
2.46
1.80
1.79
1.77
1.76
1.74
NH
N
O
CH3
1.00
1.19
1.20
3.98
2.05
3.38
3.93
2.37
2.38
f1 (ppm)

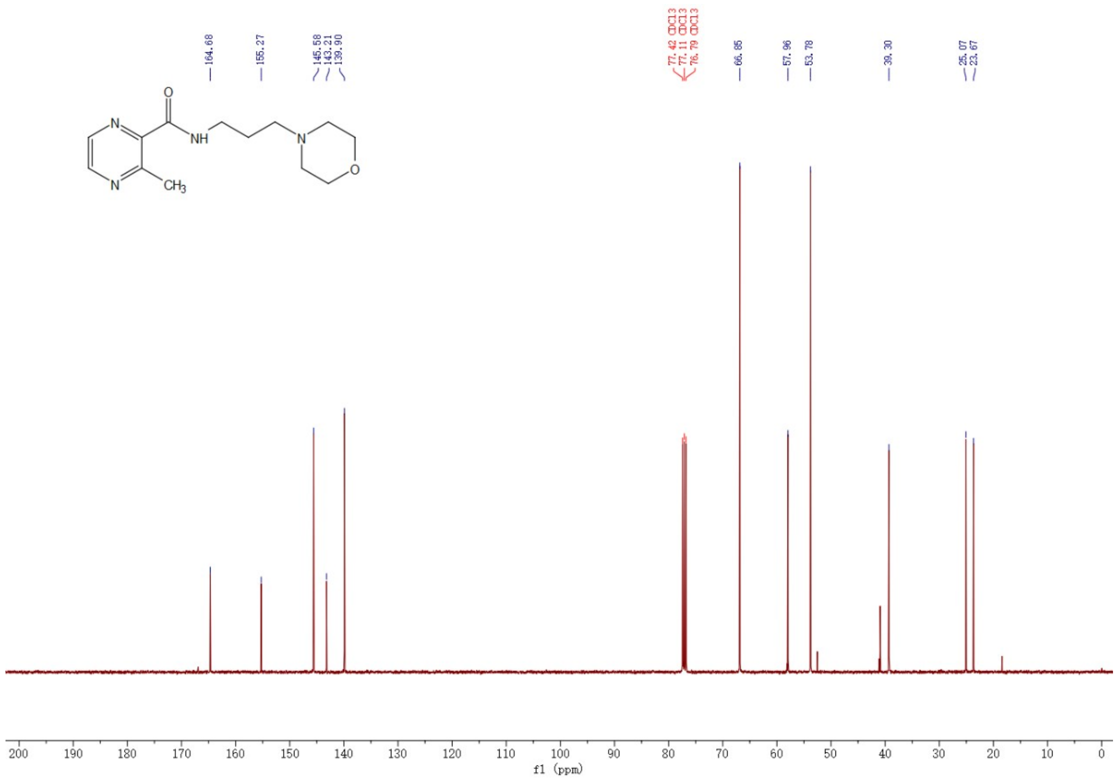
164.68
155.27
145.58
143.21
139.90
77.42 CDCl3
77.11 CDCl3
76.79 CDCl3
66.85
57.96
53.78
39.30
25.07
23.67
NH
N
O
CH3
f1 (ppm)

3v

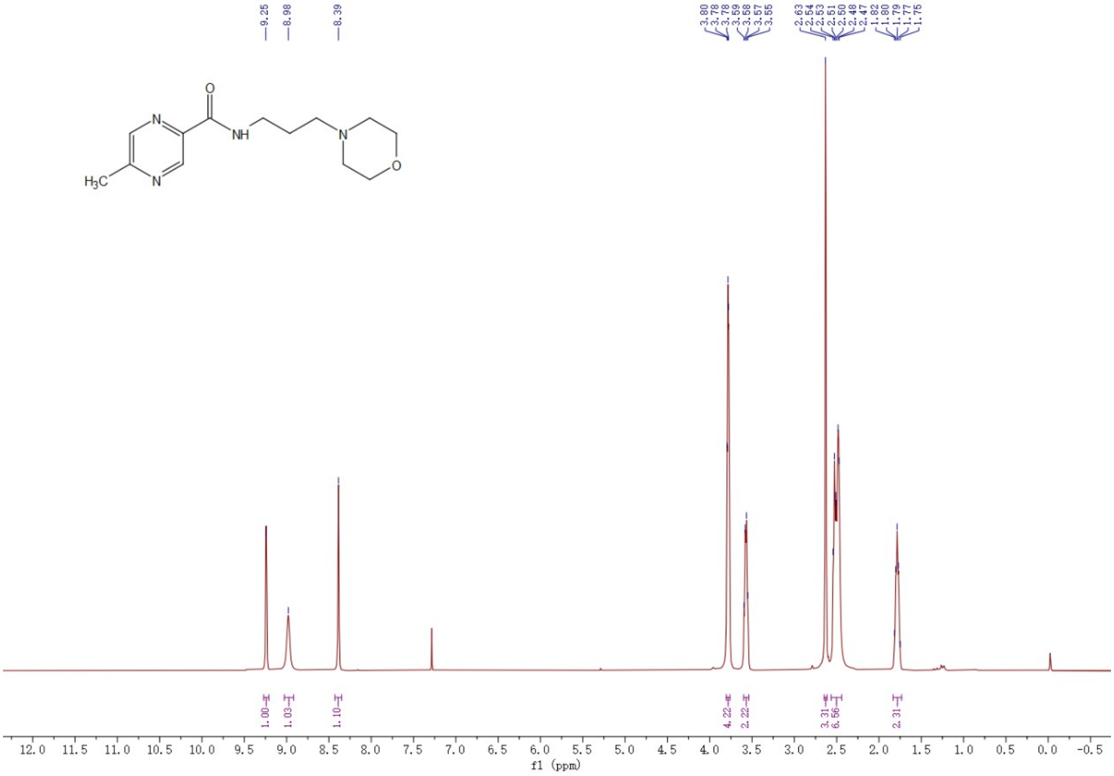

9.25
8.98
8.39
3.80
3.78
3.78
3.59
3.58
3.57
3.55
2.63
2.54
2.53
2.51
2.50
2.48
2.47
1.82
1.80
1.79
1.77
1.75
1.00
1.03
1.10
4.22
2.22
3.31
6.56
2.31
f1 (ppm)

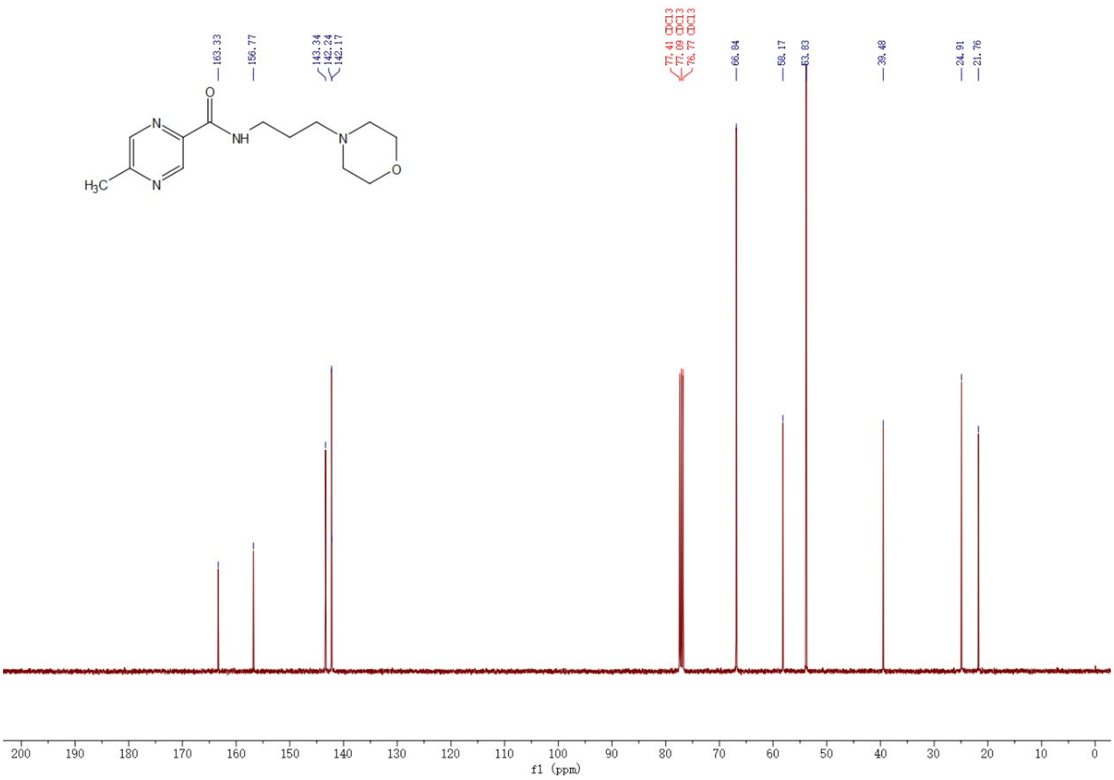

163.33
156.77
143.34
142.24
142.17
77.41 CDCl3
77.09 CDCl3
76.77 CDCl3
66.84
58.17
53.83
39.48
24.91
21.76
f1 (ppm)

3w

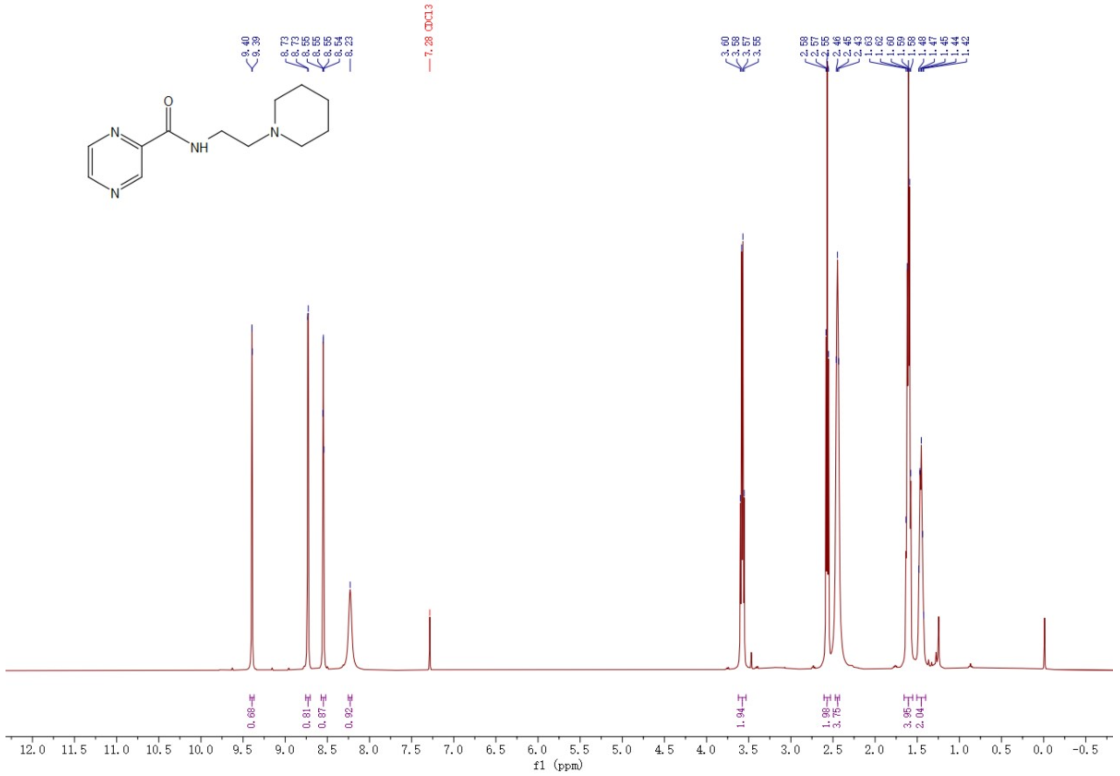
9.40
9.39
8.73
8.73
8.55
8.55
8.55
8.54
8.23
7.28 CDCl3
3.60
3.58
3.57
3.55
2.58
2.57
2.55
2.46
2.45
2.43
1.63
1.62
1.60
1.59
1.58
1.48
1.47
1.45
1.44
1.42
O
N
N
NH
N
0.68
0.81
0.87
0.92
1.94
1.98
3.75
3.95
2.04
f1 (ppm)

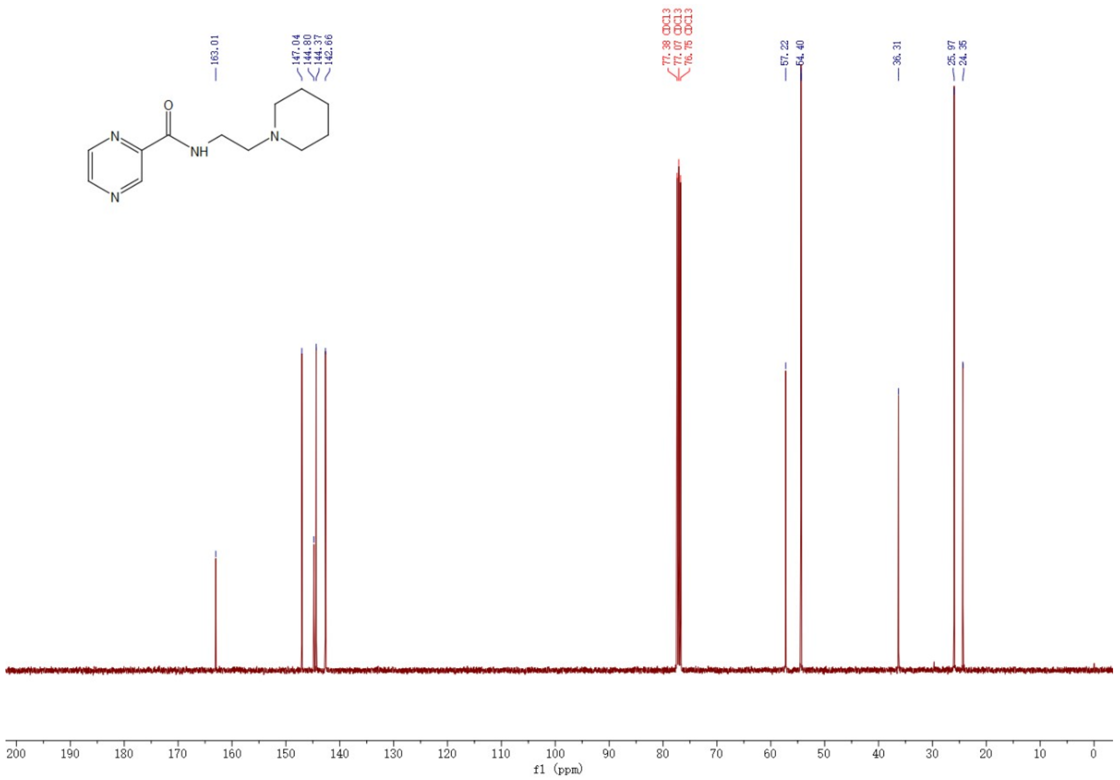
163.01
147.04
144.80
144.37
142.66
77.38 CDCl3
77.07 CDCl3
76.75 CDCl3
57.22
54.40
36.31
25.97
24.35
O
N
N
NH
N
f1 (ppm)

Notes and references

1. J. Soika, C. McLaughlin, T. Neveselý, C. G. Daniliuc, J. J. Molloy and R. Gilmour, *ACS catalysis*, 2022, **12**, 10047-10056.

2. J. Šturala, S. Boháčová, J. Chudoba, R. Metelková and R. Cibulka, *The Journal of organic chemistry*, 2015, **80**, 2676-2699.

3. E. Zukic, D. Mokos, M. Weber, N. Stix, K. Ditrich, V. Ferrario, H. Müller, C. Willrodt, K. Gruber, B. Daniel and W. Kroutil, *ACS catalysis*, 2024, **14**, 8913-8921.

4. F. A. Wati, P. U. Adyarini, S. Fatmawati and M. Santoso, *Medicinal Chemistry Research*, 2020, **29**, 2157-2163.
